# Functionalization of Gold Nanostars with Cationic β-Cyclodextrin-Based Polymer for Drug Co-Loading and SERS Monitoring

**DOI:** 10.3390/pharmaceutics13020261

**Published:** 2021-02-15

**Authors:** Orlando Donoso-González, Lucas Lodeiro, Álvaro E. Aliaga, Miguel A. Laguna-Bercero, Soledad Bollo, Marcelo J. Kogan, Nicolás Yutronic, Rodrigo Sierpe

**Affiliations:** 1 Laboratorio de Nanoquímica y Química Supramolecular, Departamento de Química, Facultad de Ciencias, Universidad de Chile, Las Palmeras 3425, Ñuñoa, Santiago 7800003, Chile; orlando.donoso@ug.uchile.cl; 2 Laboratorio de Nanobiotecnología y Nanotoxicología, Departamento de Química Farmacológica y Toxicológica, Facultad de Ciencias Químicas y Farmacéuticas, Universidad de Chile, Santos Dumont 964, Independencia, Santiago 8380000, Chile; mkogan@ciq.uchile.cl; 3 Advanced Center for Chronic Diseases (ACCDiS), Universidad de Chile and Pontificia Universidad Católica de Chile, Santiago 8380000, Chile; sbollo@ciq.uchile.cl; 4 Laboratorio de Química teórica, Departamento de Química, Facultad de Ciencias, Universidad de Chile, Las Palmeras 3425, Ñuñoa, Santiago 7800003, Chile; lucas.lodeiro@ug.uchile.cl; 5 Laboratorio de Espectroscopía Vibracional, Departamento de Química, Facultad de Ciencias, Universidad de Chile, Las Palmeras 3425, Ñuñoa, Santiago 7800003, Chile; alvceali@uchile.cl; 6 Instituto de Nanociencia y Materiales de Aragón (INMA), CSIC-Universidad de Zaragoza, 50009 Zaragoza, Spain; malaguna@unizar.es; 7 Laboratorio de Biosensores, Departamento de Química Farmacológica y Toxicológica, Facultad de Ciencias Químicas y Farmacéuticas, Universidad de Chile, Santos Dumont 964, Independencia, Santiago 8380000, Chile

**Keywords:** gold nanostars, cationic β-cyclodextrin-based polymer, phenylethylamine, piperine, drug co-loading, surface-enhanced Raman scattering

## Abstract

Gold nanostars (AuNSs) exhibit modulated plasmon resonance and have a high SERS enhancement factor. However, their low colloidal stability limits their biomedical application as a nanomaterial. Cationic β-cyclodextrin-based polymer (CCD/P) has low cytotoxicity, can load and transport drugs more efficiently than the corresponding monomeric form, and has an appropriate cationic group to stabilize gold nanoparticles. In this work, we functionalized AuNSs with CCD/P to load phenylethylamine (PhEA) and piperine (PIP) and evaluated SERS-based applications of the products. PhEA and PIP were included in the polymer and used to functionalize AuNSs, forming a new AuNS-CCD/P-PhEA-PIP nanosystem. The system was characterized by UV–VIS, IR, and NMR spectroscopy, TGA, SPR, DLS, zeta potential analysis, FE-SEM, and TEM. Additionally, Raman optical activity, SERS analysis and complementary theoretical studies were used for characterization. Minor adjustments increased the colloidal stability of AuNSs. The loading capacity of the CCD/P with PhEA-PIP was 95 ± 7%. The physicochemical parameters of the AuNS-CCD/P-PhEA-PIP system, such as size and Z potential, are suitable for potential biomedical applications Raman and SERS studies were used to monitor PhEA and PIP loading and their preferential orientation upon interaction with the surface of AuNSs. This unique nanomaterial could be used for simultaneous drug loading and SERS-based detection.

## 1. Introduction

Gold nanoparticles are employed in bionanotechnology because they exhibit excellent optoelectronic properties and high surface reactivity, which enable preparation of biocompatible nanomaterials with specific functions [1,2,3,4]. Bioapplications of gold nanoparticles include targeted therapy, molecular delivery, imaging, detection, and theranostics [5,6,7]. Current studies of gold nanoparticles have focused on their anisotropic forms because adjustments of the size, morphology and other properties can improve specific characteristics of these nanoparticles. The types of gold nanoparticles include nanorods [8,9,10], nanoprisms [11,12], nanocubes [13,14], and nanostars [15,16,17,18].

Gold nanostars (AuNSs) are of special interest because morphological modifications, such as number of tips and their length, can be used to tune the position of the band of localized surface plasmon resonance [19,20] to place it within a biological window [21,22]. In addition, strong electric field enhancements due to the presence of multiple sharp tips of AuNSs transform them into high-performance detection materials for surface-enhanced Raman scattering (SERS) [23,24] and surface-enhanced fluorescence (SEF) [25,26]. Properly designed AuNSs can be applied for drug delivery and/or photothermal therapy in the treatment of diseases [16,18] and can be combined with diagnostic mechanisms for surface enhancement in imaging or detection [15,22,26].

An increase in the reactive surface area due to specific morphology of AuNSs causes a substantial decrease in colloidal stability [27,28,29]. Multiple strategies have been tested to increase the stability and prevent the aggregation of this nanomaterial, such as stabilizing agents containing thiol or amine groups [30,31]. The stabilizing agents may also have specific new functions, such as detection, therapy, or vectorization. Potential agents include a wide variety of biomolecules, such as peptides [32], proteins [33,34], genetic material [35,36], antibodies [37,38], drugs [39,40], Raman reporters [41,42], fluorophores [26,43], polymers [44,45,46], and supramolecular complexes [47].

β-Cyclodextrin (βCD) is a water-soluble nontoxic cyclic oligosaccharide formed by 7 linked units of glucopyranose with a bucket-like shape; this compound has various biomedical applications and is approved by the FDA. The βCD cavity is partially hydrophobic and can thus incorporate nonpolar species of appropriate dimensions to form an inclusion complex with increased solubility and protection against oxidation, enzyme-mediated degradation or photolysis [48,49]. Due to cavity dimensions, βCD is the most commonly used compound for biological applications, especially drug delivery [50,51]. Additionally, βCD has been successfully used in association with gold nanoparticles, forming the systems with potential applications in drug transport [52,53,54,55]. However, βCD has lower solubility than that of α- or γ-cyclodextrins. These factors and cytotoxicity related to βCD binding to and extraction of cell membrane cholesterol limit the use of βCD [56,57]. Various cyclodextrin derivatives have been investigated to overcome these drawbacks [58,59,60]. These derivatives include new polymeric forms that were shown to have improved aqueous solubility and transport efficiency and increased loading capacity for several drugs [61,62,63,64,65,66]. Notably, βCD-based polymers modified with quaternary ammonium groups were shown to be ineffective in hosting cholesterol [65,67]. Species with quaternary ammonium group have been extensively used as stabilizers of AuNPs [68,69,70,71]. The interaction of these groups with AuNPs is electrosteric due to electrostatic interactions between anions (commonly chloride or bromide) and cations (quaternary ammonium) on the surface of AuNPs and steric hindrance provided by the organic chain of the species containing quaternary ammonium [72,73,74,75]. Thus, the cationic groups present in the polymer are responsible for stabilization of AuNSs, leaving multiple βCD cavities of the polymer free to form inclusion complexes with one or more species through the supramolecular soft-bond chemistry that defines the host–guest interactions [76,77].

In this work, we synthesized AuNSs using a simple and surfactant-free method. The prepared nanoparticles produce an absorbance band located within a biological window. Subsequently, AuNSs were functionalized with a cationic cyclodextrin-based polymer (CCD/P) to increase their stability over time, forming a new nanomaterial, AuNS-CCD/P. Phenylethylamine (PhEA) and piperine (PIP) were incorporated in the system to evaluate drug co-loading capacity and provide a proof-of-concept of the potential SERS application of the AuNS-CCD/P system.

PhEA is a psychoactive stimulant employed as an antidepressant and does not induce tolerance. However, PhEA is rapidly metabolized in the body by the MAO-B enzyme and thus cannot be accumulated at sufficient concentrations in the brain [78,79]. PIP is a component of black pepper used as an inhibitor of MAO-A and MAO-B [80,81] that has antibacterial and insecticidal activity [82], activates the sympathetic system [83] and facilitates lipolysis in white adipose tissue [84].

This study may contribute to the development of nanotechnology systems based on AuNSs and CCD/P; these materials have not been reported previously. The new AuNS-CCD/P system can be used for simultaneous drug loading and in SERS-based detection.

## 2. Materials and Methods

### 2.1. Materials

Tetrachloroauric acid (HAuCl_4_*3H_2_O) ≥ 99.9%, molar weight: 393.83 g/mol; βCD (C_42_H_70_O_35_) ≥ 98%, 1134.98 g/mol; (±) epichlorohydrin (EP, C_3_H_5_ClO) ≥ 99%, 92.52 g/mol; choline chloride (CC, C_5_H_14_ONCl) ≥ 98%, 139.62 g/mol; PhEA (1-phenylethan-2-amine, C_8_H_11_N) ≥ 99%, 121.18 g/mol, δ: 0.962 g/mL; PIP ((2E,4E)-5-(2H-1,3-benzodioxol-5-yl)-1-(piperidin-1-yl)penta-2,4-dien-1-one, C_17_H_19_NO_3_) ≥ 97%, 285.3 g/mol; and sodium hydroxide (NaOH) ≥ 97%, 40.00 g/mol were provided by Sigma Aldrich (Saint Louis, MO, USA). Hydroxylamine hydrochloride (NH_2_OH*HCl) ≥ 98%, 69.49 g/mol; hydrochloric acid (HCl) for analysis, 36.46 g/mol; and water (nanopure) were provided by Merck (Darmstadt, Germany).

### 2.2. Synthesis of Gold Nanostars

AuNSs were synthesized according to a protocol reported by Minati et al. [85] with small adjustments to increase colloidal stability. One milliliter of 100 mmol/L hydroxylamine solution was adjusted to pH 12.3 with NaOH. Subsequently, 100 µL of 10.0 mmol/L HAuCl_4_ was added to the solution, under strong and constant agitation. The final concentration ratio of hydroxylamine/Au^3+^ was 100. An instant color change to deep blue indicated that the reaction was successful. One minute later, the solution was diluted three-fold to increase colloidal stability in water. Colloidal solution was naturally decanted for approximately 8 h to form a reversible agglomerate, which was dispersed by sonication, recovering its color and properties. All procedures were performed at 25 °C.

### 2.3. Synthesis of Cationic β-Cyclodextrin-Based Polymer

CCD/P was synthesized according to the protocol described by Li et al. [58]. Initially, 3.5 mL of aqueous 2.7 mol/L NaOH solution was mixed with 994 mg of βCD inside a round-bottom distillation flask. A water bath was mounted on an iStir HP550P heating plate, with magnetic stirring. The flask with the solution was placed inside a water bath at 25 °C and incubated under gentle agitation for 24 h. Then, 125.2 mg of CC was rapidly added. Subsequently, 1040 µL of EP was added at a flow rate of 30 µL/min for 35 min. Then, the temperature of the solution was increased to 60 °C, and the mixture was stirred at 52× *g* for 2 h. The molar ratio of βCD/EP/CC used for the synthesis was 1/15/1. The reaction was stopped by neutralization with aqueous 3 mol/L HCl solution. The obtained solution was dialyzed for 24 h. Finally, the resulting polymer was lyophilized to completely remove the solvent.

### 2.4. Inclusion of Phenylethylamine and Piperine in Cationic β-Cyclodextrin-Based Polymers

The synthesis of CCD/P-PhEA-PIP was performed using 200 mg of CCD/P, 11.7 µL of PhEA and 25.14 mg of PIP to achieve a molar ratio of 1/0.5/0.5 since the βCD-PhEA and βCD-PIP complexes have a 1:1 stoichiometric ratio [55,86]. Aqueous solution of CCD/P (3 mL) and ethanol solution of PIP (0.5 mL) were prepared. The drug and CCD/P solutions were slowly mixed and incubated at room temperature for 24 h. These conditions favored gradual inclusion of both compounds to ensure high occupancy of the hydrophobic cavity of βCD. Finally, the solution was dialyzed for 24 h to remove unincorporated drug molecules and lyophilized to obtain the polymer with PhEA and PIP in solid state. All procedures were performed at 25 °C.

### 2.5. Functionalization of Gold Nanostars with Cationic β-Cyclodextrin-Based Polymer Loaded with Phenylethylamine and Piperine

For functionalization with CCD/P-PhEA-PIP, the AuNSs were initially agglomerated, and the supernatant was removed; subsequently, 1.0 mg of CCD/P-PhEA-PIP dissolved in 3.0 mL of water was added. Then, the system was resuspended by sonication for 3 min. This procedure was repeated two more times; finally, the AuNS-CCD/P-PhEA-PIP system was resuspended in water. All procedures were performed at 25 °C.

### 2.6. Characterization of the Systems

AuNSs, CCD/P, CCD/P-PhEA-PIP, AuNS-CCD/P, and AuNS-CCD/P-PhEA-PIP were characterized using the following approaches.

UV–VIS spectroscopy was performed at 25 °C using a UV 2450 spectrophotometer (Shimadzu, Kyoto, Japan) with water as the baseline. Zeta potential and dynamic light scattering analyses were performed at 25 °C using a Zetasizer (Nano ZS model, Malvern, Malvern, UK). The size distribution of the samples was determined based on the results of the intensity distribution values using the cumulant method. The Smoluchowski approximation was used to calculate the Z potentials based on the measured electrophoretic mobility. Field emission scanning electron microscopy (FE-SEM) was performed using a Leo Zeiss Supra 35VP microscope at the acceleration voltages of 15 kV and 2 kV. Transmission electron microscopy (TEM) was performed using a JEOL 2000FX TEM microscope at 200 kV to determine the average particle size and distribution of the samples. The average particle size was calculated using Digital Micrograph software. ^1^H-NMR was performed using a Bruker Advance 400 MHz superconducting NMR spectrometer at 300 K, and all solid samples were dissolved in DMSO-d6 (99.99% D). The precursors EP, CC, βCD, PhEA, and PIP and the samples of CCD/P and CCD/P-PhEA-PIP were measured directly; the samples of AuNS-CCD/P (10 mg) and AuNS-CCD/P-PhEA-PIP (10 mg) were lyophilized and then measured. FT/IR spectroscopy was performed using a spectrometer (model FT/IR-4600, Jasco, Easton, MD, USA). Thermogravimetric analysis (TGA) was performed using an instrument (model 4000, Perkin-Elmer, Waltham, MA, USA). The temperature of the furnace was programmed to increase at a rate of 10 °C/min from 30 °C to 800 °C under an atmosphere of N_2_ (a flow rate of 20 mL/min). Mass spectrometry was performed using a MALDI-TOF Microflex (Bruker Daltonics Inc., Billerica, MA, USA) in the positive and negative ion mode by linear detection in the presence of the DHB matrix. Entrapment efficiency and loading capacity were determined using an SPR instrument (Dual channel SR7500DC, Reichert Technologies, Depew, NY, USA) with an autosampler system. Data acquisition was performed using Integrated SPR Autolink (Reichert Technologies, Depew, NY, USA). The data were processed using TraceDrawer 1.6.1 and OriginPro 8.0 software.

### 2.7. Raman and Surface-Enhanced Raman Scattering Measurements

The Raman and SERS measurements were performed using a micro-Raman RM 1000 spectrometer (Renishaw, Wotton-under-Edge, UK) equipped with lasers at 514, 633, and 785 nm. The apparatus was coupled to a DM LM microscope (Leica Microsystems, Wetzlar, Germany) and an electrically cooled CCD camera (Renishaw, Wotton-under-Edge, UK). The Raman signal was calibrated to the 520 cm^−1^ line of silicon using a lens with a 50× objective. The laser power incident on the sample was approximately 0.2 mW. The acquisition time was set between 10 and 20 s per accumulation; the average number of accumulations was 10, with a spectral resolution of 4 cm^−1^. Data were collected between 0 and 3000 cm^−1^, and the recorded spectral region was between 200 and 2000 cm^−1^. The conditions of spectral recording and laser wavelength were selected to avoid the degradation and possible fluorescence of the samples; accordingly, the 785 nm laser line was used for SERS and Raman spectral scanning. Raman spectra of PhEA were measured in samples prepared by drying of multiple drops of the solution on silica. The Raman spectra of PIP, CCD/P, and CCD/P-PhEA-PIP were collected in the solid state. The SERS spectra of AuNS-CCD/P and AuNS-CCD/P-PhEA-PIP were obtained in aqueous solution supported on silica.

### 2.8. Molecular Model, Methods, and Calculations for Theoretical Raman Spectra

All calculations were performed using density functional theory (DFT) in Gaussian09, revision D.01 [87], on a high-performance computing (HPC) cluster. The B3LYP [88,89] hybrid exchange-correlation functional and 6–311*G(d,p) basis set were used. B3LYP is a functional suitable for organic molecules [90,91] and is widely used in this field of research to assign vibrational signals [55,92]. A Gaussian valence triple Z basis set with polarization and diffuse orbitals for heavy atoms and polarization orbitals for hydrogen atoms was selected to achieve a good system description with a low basis set incompleteness.

The first step included molecular geometry optimization. Scans over various angles were performed to identify various local minima. The lowest local minima geometry was used for each molecule in the gas phase. Additionally, a harmonic vibrational frequency calculation was performed to verify that the optimized geometries were genuine minima in the potential energy surface. No symmetry or geometry restrictions were imposed during the optimizations. The second step included an anharmonic vibrational frequency and Raman intensity calculations, through second-order vibrational perturbation theory (VPT2) [93], for previously relaxed structures to obtain a set of vibrational signals for Raman spectra and their normal modes. This information was used to assign the normal modes of vibration to experimental Raman data through atomic displacement vectors and direct visualization.

## 3. Results and Discussion

### 3.1. Preparation and Stabilization of Gold Nanostars

**Figure 1:**
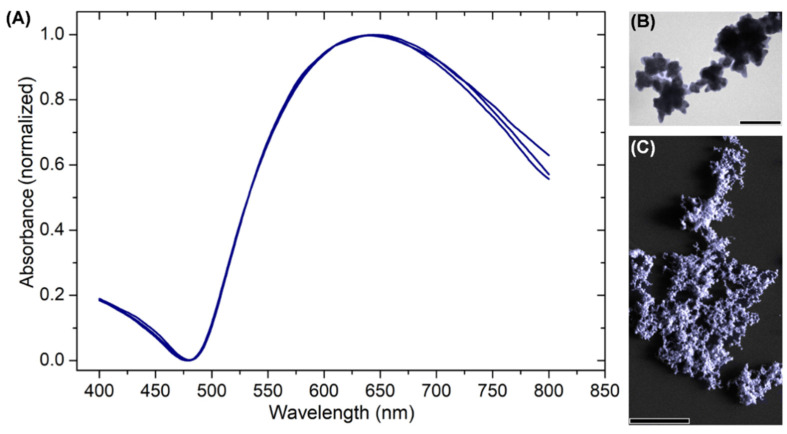
(**A**) AuNSs absorbance spectrum in triplicate; (**B**) and (**C**) TEM and FE-SEM images of AuNSs, respectively. Scale bars: (**B**) 100 nm, (**C**) 2 µm.

Successful production of AuNSs was confirmed using UV–VIS spectroscopy; this technique allows the evaluation of optical properties of nanoparticles by providing preliminary information on the size, shape, or state of aggregation, which are associated with the surface plasmon resonance characteristics of the nanoparticles [94,95]. Figure 1A shows the spectra with a maximum absorbance at 639 nm and a plasmon bandwidth of 275 nm characteristic for AuNSs prepared by this method. Additionally, the TEM and FE-SEM images shown in Figure 1B,C revealed that AuNSs had a central spherical morphology with multiple short arms [85,96]. The shoulders were not observed, demonstrating the absence of by-product nanospheres.

Minati et al. [85] reported that AuNSs obtained using hydroxylamine as a reducing agent were stable for 3 h without other stabilizers; this synthesis was fast and was accomplished in less than a minute; however, the procedure does not control particle size and cannot produce particles with longer stabilization time. Various minor adjustments to the synthesis procedure were studied to increase colloidal stability, expand the functionalization alternatives and allow the use of generated nanomaterials. Prepared AuNSs were immediately diluted in water to decrease particle concentration and avoid the aggregation caused by the Brownian movement. This strategy increased the stability time to at least seven days and was a fast, simple, and effective means to avoid coating the surface of the material with stabilizing molecules that may limit surface reactivity and subsequent use of the particles.

**Figure 2:**
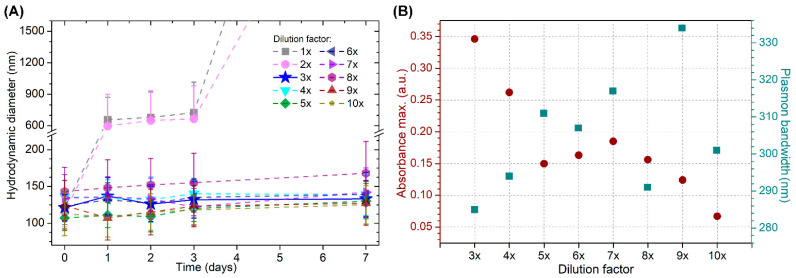
(**A**) Hydrodynamic diameter vs. time elapsed from the synthesis of AuNSs at various dilution factors from 1× to 10×; (**B**) absorbance maxima (left) and plasmon bandwidths (right) of AuNSs in colloidal solution at various dilution factors.

The tested dilution factors ranged from 2× to 10×, and 3×–10× dilutions resulted in stable AuNSs. Figure 2 shows the changes in the hydrodynamic diameter versus time (Figure 2A) and the absorbance maxima and plasmon bandwidths of the spectra (Figure 2B) of AuNSs at various dilutions. The greatest absorbance maximum and lowest plasmon bandwidth were observed in a sample with a 3× dilution factor, indicating a higher concentration and smaller size distribution than those in other samples; this dilution factor was therefore used in the optimized procedure. At this dilution, AuNSs gradually agglomerated at the bottom of the vial. This process was completely reversed by sonication.

The strategy used to resuspend AuNSs is not trivial. Thus, two methods to resuspend previously agglomerated AuNSs were compared: sonication and mechanical agitation. The surface charges of the resuspended colloidal solutions were −49 (±3) mV in the case of sonication and −55 (±2) in the case of mechanical agitation. The hydrodynamic diameters were 121 (±18) nm in the first method and 249 (±49) nm in the second method, and polydispersity indices were 0.22 and 0.34, respectively. The UV–VIS spectra showed an increase in absorbance, a hypsochromic shift and a lower plasmon bandwidth in particles obtained by sonication. Therefore, the mechanical agitation method was not sufficient to disaggregate colloidal solution after agglomeration, while the sonication method achieved efficient resuspension. Moreover, comparison of these characteristics with the properties of AuNSs in the colloidal solution immediately after synthesis indicated that sonication can enrich AuNSs. Thus, sonication was used for functionalization of the surface of AuNSs with CCD/P and for washing.

**Figure 3:**
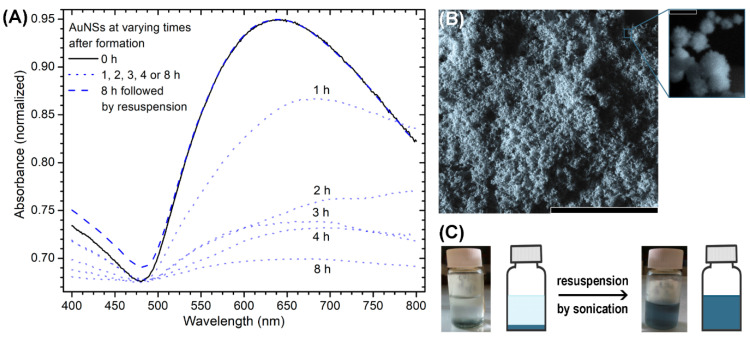
(**A**) Absorbance spectra of 3×-diluted AuNSs during agglomeration at various time points and after subsequent resuspension; (**B**) SEM micrographs and (**C**) scheme of agglomeration of 3×-diluted AuNSs and 3×-diluted AuNSs resuspended using sonication. Scale bars: (**B**) 5 µm, zoomed image: 100 nm.

Figure 3A shows the loss of plasmon resonance of AuNSs over 8 h due to agglomeration and the recovery of the plasmon resonance signal to the initial value after subsequent resuspension by sonication. Figure 3B shows the images and FE-SEM micrographs of the solution after agglomeration and resuspension, demonstrating that AuNSs did not lose their morphology or surface and optical properties. This process can be repeated for three consecutive days (for additional details and results, see the supplementary material, Section S1).

### 3.2. Preparation of Cationic β-Cyclodextrin-Based Polymers and Loading with Phenylethylamine and Piperine

**Figure 4:**
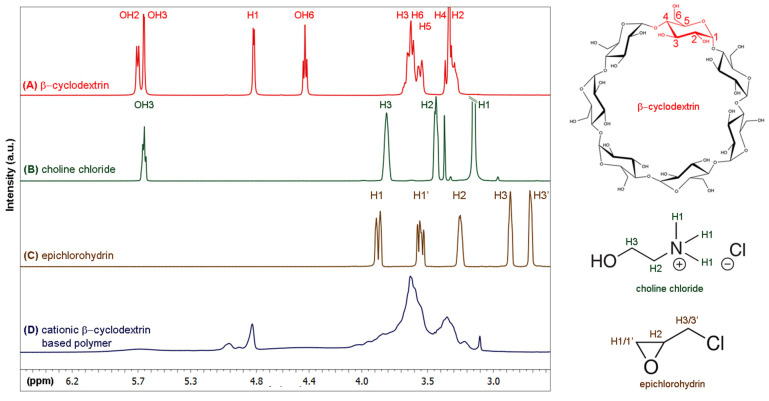
^1^H-NMR spectra of (**A**) β-cyclodextrin, (**B**) choline chloride, (**C**) epichlorohydrin, and (**D**) cationic β-cyclodextrin-based polymer and their molecular structures and proton assignments (left).

AuNSs were stabilized and functionalized with CCD/P and the PhEA and PIP drugs to construct a new nanosystem for drug co-loading. Initially, CCD/P was synthesized according to the protocol of Li et al. [58] via basic reaction of EP with CC and βCD. CCD/P formation was confirmed using ^1^H-NMR spectroscopy. Figure 4 shows the spectra and scheme of the assigned structures of (A) βCD, (B) CC, and (C) EP and the spectrum of (D) CCD/P. Noticeable changes in the signals corresponding to the hydroxyl groups of βCD and CC were induced by polymerization of the compounds. Additional chemical shifts in the H3/H3’ protons of EP (used as a linker) were detected. A widening of the signals assigned to the internal protons of βCD and protons participating in the ether-type bonds in the region between 3.5 and 3 ppm was detected as a result of the formation of the polymer [97].

CCD/P was also characterized by IR spectroscopy and mass spectrometry. In the IR spectrum, the attenuation of the band near 3600 cm^−1^ corresponded to the stretching of the OH groups, and the changes in the interval from 1050 to 1200 cm^−1^ corresponded to the stretching of the ether bonds, suggesting the formation of the ether bonds by the epoxide moiety of EP attacking hydroxyl groups of CC and βCD [98] in agreement with the NMR results. The mass spectrum detected variable degrees of polymerization of up to 14 βCD units in each chain (see figures in the Supplementary Material, Section S2.1).

**Figure 5:**
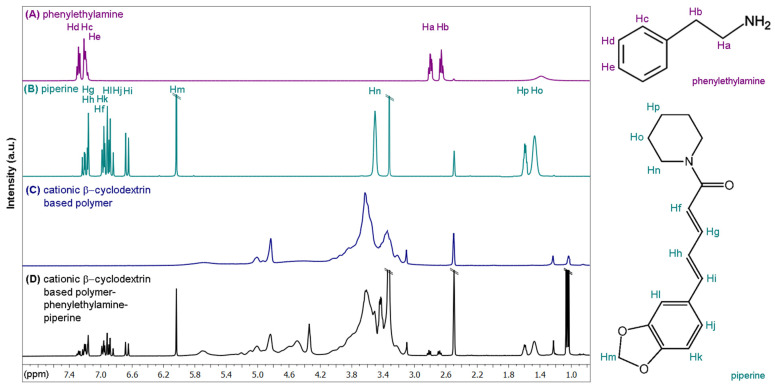
^1^H-NMR spectra of (**A**) phenylethylamine, (**B**) piperine, (**C**) cationic β-cyclodextrin-based polymer, and (**D**) cationic β-cyclodextrin-based polymer loaded with phenylethylamine and piperine; the molecular structures and proton assignments of the drug molecules are shown on the left.

The PhEA and PIP drugs were used to evaluate the drug inclusion capacity of the polymers. The resulting loaded CCD/P was used for surface modification of AuNSs. The inclusion process was characterized using ^1^H-NMR. Figure 5 shows the spectra of PhEA, PIP and CCD/P (A, B, and C, respectively) and the assigned structures of PhEA and PIP. The spectrum (D) shows the signals of the polymer and both drugs, demonstrating simultaneous inclusion of PhEA and PIP in the cavities of polymerized βCD, forming a new CCD/P-PhEA-PIP system.

Integration of the signals corresponding to each drug in the CCD/P-PhEA-PIP system showed a stoichiometric relationship corresponding to three-fold higher inclusion of PIP than that of PhEA. Furthermore, integration of signals corresponding to βCD cavities of CCD/P in the CCD/P-PhEA-PIP system showed a stoichiometric relationship corresponding to 4:1:3 βCD: PhEA:PIP, suggesting strongly that the inclusion of the drugs occurred exclusively through the βCD cavities in a 1:1 ratio (additional details are shown in the supplementary material, Section S2.2). The association constants for βCD-PhEA and βCD-PIP at a 1:1 stoichiometry in aqueous solution were previously reported to be 760 M^−1^ [55] and 3244 M^−1^ [99], respectively. These results may explain preferential inclusion of PIP in βCD.

The NMR spectra provided the details of the inclusion process based on the chemical shifts of free PhEA and PIP and compounds interacting with the CCD/P cavities. Table 1 shows the proton assignments for the drugs and the corresponding chemical shifts. The assignments were based on the ^1^H-NMR studies of PhEA and PIP [55,100]. In the latter case, adjustments were used to accurately identify all signals. The proton signals of the aryl group of PhEA (Hc, Hd, and He) showed chemical shifts towards higher frequencies, and the proton signals of the ethylamine chain (Ha, Hb) showed chemical shifts towards lower frequencies. This result suggested that the aryl group of PhEA was completely incorporated in the βCD cavity, and the ethylamine chain was close to one of the apertures of the matrix and interacted with the hydroxyl groups, as reported previously for the βCD-PhEA complex [55].

Hp, Ho, Hn, and Hf of PIP showed no displacement, and the Hi, Hh, Hg, Hj, Hk, and Hm protons showed a chemical shift to a high frequency, indicating dynamic inclusion by the benzodioxol and ethylene regions, as reported in a previous study on the βCD-PIP complexes [86,99,100].

Additionally, CCD/P-PhEA-PIP was studied using FT-IR spectroscopy. A major contribution of the polymer signals was observed in the spectrum (see Figure in the Supplementary Material, Section S2.3). Therefore, Raman spectroscopy provided complementary and valuable information on the interaction of the drugs included in CCD/P.

**Figure 6:**
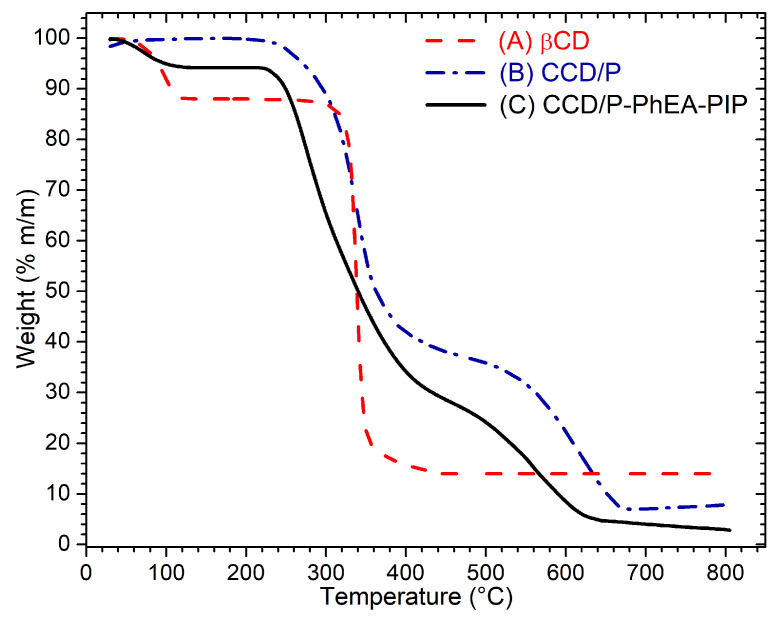
Thermogravimetric decomposition curves of (**A**) βCD, (**B**) CCD/P, and (**C**) CCD/P-PhEA-PIP in a temperature range between 10 and 800 °C in an atmosphere of N_2_.

The CCD/P and CCD/P-PhEA-PIP systems were also characterized using TGA to obtain the information on the formation of the polymers and effective inclusion of the drugs within the polymer matrix based on the changes in their thermal decomposition [101,102]. Figure 6 shows the thermal decomposition curves of native βCD (A), CCD/P (B), and CCD/P-PhEA-PIP (C).

Comparison with the thermogram of native βCD indicated fundamental differences that confirmed CCD/P formation. Specifically, the βCD thermogram showed the first decomposition of 10% of mass between 0 and 100 °C attributed to the loss of water molecules; this weight loss was not observed in the polymer because the synthesis method included the removal of water. The second range of the decomposition of βCD was observed between 300 and 368 °C, corresponding to the loss of 69% of the sample mass. The CCD/P thermogram detected two decomposition ranges from 220 to 406 °C and from 497 to 683 °C, corresponding to the loss of 61% and 20% of the sample mass, respectively. The peaks of the derivative curves for CCD/P were detected at 339 and 625 °C (see the Supplementary Material, Section S2.3). This result was due to an increase in the decomposition temperature of the polymer chains caused by the matrix that corresponded to an additional displacement peak.

The CCD/P-PhEA-PIP thermogram shows the changes in the decomposition curve compared with the CCD/P and βCD thermograms. The loss of 6% of the sample mass was observed at 100 °C. This mass loss was due to the inclusion of PhEA and PIP in the polymers in aqueous solution, which caused the inclusion of water molecules in the cavities. The second decomposition from 220 to 450 °C resulted in the loss of 61% of the sample mass and corresponded to a peak at 273 °C in the derivative curve. The final decomposition from 464 to 650 °C corresponded to a peak at 567 °C in the derivative curve. An expansion of the range of the second decomposition and an increase in the total mass loss of the sample by 7% were attributed to the presence of PhEA and PIP. This effect is typically observed when βCD cavities harbor the guest molecules [103,104]. Therefore, various techniques confirmed effective inclusion of PhEA and PIP in CCD/P.

**Figure 7:**
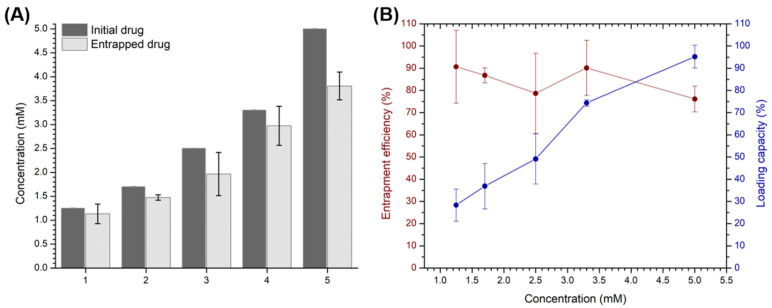
(**A**) Bar graph comparing the initial concentration of PhEA-PIP and the concentration of PhEA-PIP entrapped in the CCD/P in five different assays (*n* = 3). (**B**) Percentages of entrapment efficiency (left) and loading capacity (right) of CCD/P with both drugs, at different concentrations.

Entrapment efficiency and loading capacity were studied using SPR, considering that the βCD cavities in the polymer are the only hydrophobic sites with adequate dimensions to include PhEA and PIP. The studies were performed at the concentrations of PhEA-PIP from 1.25 to 5.0 mM. The results are shown in the graphs in Figure 7A,B. Entrapment efficiency was decreased concomitant to an increase in the concentrations of included drugs and varied between 91 and 76%. The loading capacity was 95% at the maximum concentration of the drugs added to the system (5.0 mM initial concentration), and this concentration resulted in a 1:1 molar ratio of the βCD moiety of the polymer and the drugs. The loading capacity was decreased when lower drug concentrations were used since the resulting stoichiometric ratios were 1:>1 (additional details are shown in the Supplementary Material, Section S2.3).

### 3.3. Stabilization of Gold Nanostars with Cationic β-Cyclodextrin-Based Polymer and with Cationic β-Cyclodextrin-Based Polymer Including Phenylethylamine and Piperine

Confirmation of effective inclusion of the drugs in CCD/P enabled the formation of the AuNS-CCD/P nanosystem, covering the surface of anisotropic nanoparticles with cationic polymer. The interaction between the gold atoms and quaternary ammonium groups was demonstrated using SPR (see the Supplementary Material, Section S3.1).

Additionally, this system was simultaneously loaded with two drugs, PhEA and PIP, forming a new AuNS-CCD/P-PhEA-PIP nanosystem. Both systems were characterized using UV–VIS spectroscopy, dynamic light scattering, zeta potential analysis and TEM to evaluate various parameters, such as dimensions, morphology, absorption within a biological window and surface charge, relevant for future biomedical applications [105,106]. The results are summarized in Table 2.

In general, the changes in the plasmon resonance due to functionalization of the surface of gold nanoparticles were detected based on the UV–VIS spectra and demonstrated that AuNSs were very sensitive to these effects. Interactions with CCD/P and CCD/P-PhEA-PIP caused hypsochromic shifts of absorbance maxima by 29 and 21 nm, respectively, and a decrease in the average bandwidth by approximately 30 nm. Consequently, an increase in the hydrodynamic diameter of AuNSs from 121 nm to 167 nm was induced by functionalization with CCD/P, and an increase up to 178 nm was induced by functionalization of AuNSs with CCD/P-PhEA-PIP. The surface charge of the AuNSs was −49 mV; this highly negative value resulted in electrostatic repulsion insufficient for stabilization of anisotropic nanoparticles for more than 2 h. Functionalization of AuNSs with CCD/P and CCD/P-PhEA-PIP caused a decrease in the surface charge to approximately −15 mV in both systems due to the interactions with quaternary amino groups (with charge 1+) of the polymer. Therefore, high colloidal stability (over 7 days) of the AuNS-CCD/P and AuNS-CCD/P-PhEA-PIP systems was due to the steric impediment provided by the polymer and the drugs loaded into the polymer, which inhibited the aggregation of the nanostructures.

Additionally, ^1^H-NMR and UV–VIS spectra of the AuNS-CCD/P and AuNS-CCD/P-PhEA-PIP systems demonstrated that the presence of AuNSs did not influence the inclusion and stability of CCD/P loaded with PhEA and PIP (see the results of ^1^H-NMR and UV–VIS spectroscopy in the Supplementary Material, Sections S3.1 and S3.2).

**Figure 8:**
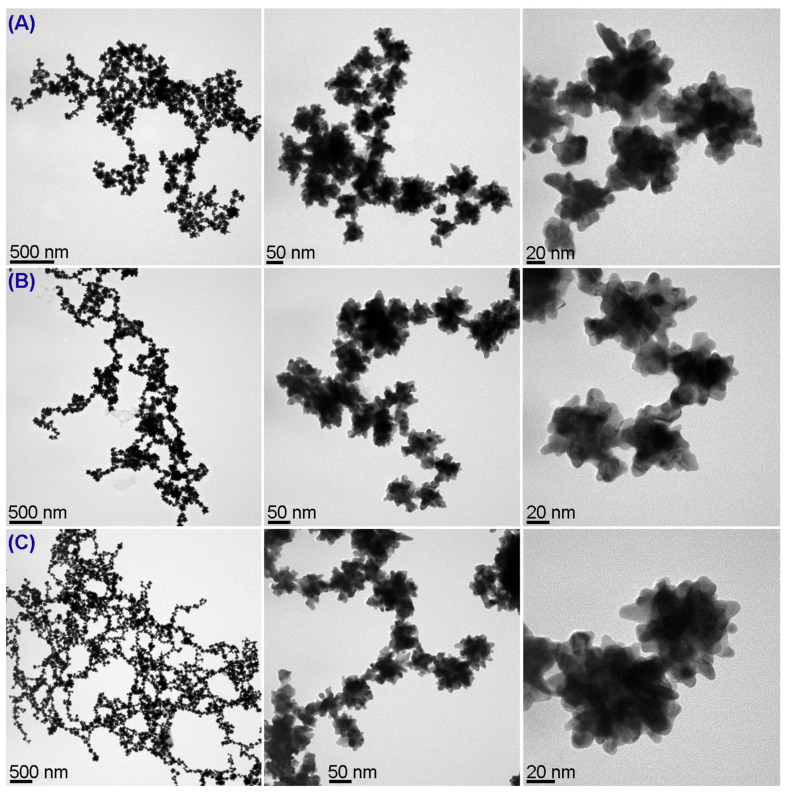
TEM micrographs of (**A**) AuNSs, (**B**) AuNS-CCD/P, and (**C**) AuNS-CCD/P-PhEA-PIP at an acceleration voltage of 200 kV.

Figure 8 shows the TEM images of (A) AuNSs, (B) AuNSs covered with CCD/P, and (C) AuNSs covered with CCD/P-PhEA-PIP (see histogram in the Supplementary Material, Section S3.3). These TEM images demonstrated that functionalization with CCD/P and subsequent inclusion of PhEA and PIP did not cause aggregation of the colloidal system, which maintained its size and characteristic morphology. The calculated sizes considered only the diameter of AuNSs, since Au has a higher electron density than the organic material (polymers and drugs) that covers the particles; therefore, the organic structures were not observed by TEM. Thus, the variations in the average size (last column of Table 2) were within the range of the standard deviation.

Surface characterization demonstrated effective interactions between AuNSs and CCD/P in solution due to the negative charge of the gold atoms on the surface and the quaternary ammonium groups of the polymer, displacing their electric charge towards neutrality and forming a stable colloidal system with steric hindrance and an average size of the drug-loaded nanosystem of 78 ± 23 nm. The relevant factors provided new biomedical functions to highly anisotropic nanomaterials without the addition of toxic reagents or loss of the optoelectronic properties.

An increase in colloidal stability of AuNSs was achieved due to minor adjustments in the synthesis and to functionalization with CCD/P. Furthermore, loading the new nanomaterial with two synergistic drugs (PhEA and PIP) may enable future studies aimed at applications in multitherapy, drug delivery, or theranostics. Based on these results, we continued the investigation of the surface using SERS to contribute to the development of applications of the AuNS-CCD/P system based on monitoring of the species proximal to the surface of the particles.

### 3.4. SERS Measurements of Phenylethylamine and Piperine Included on the Surface of Cationic β-Cyclodextrin-Based Polymer-Functionalized Gold Nanostars

**Figure 9:**
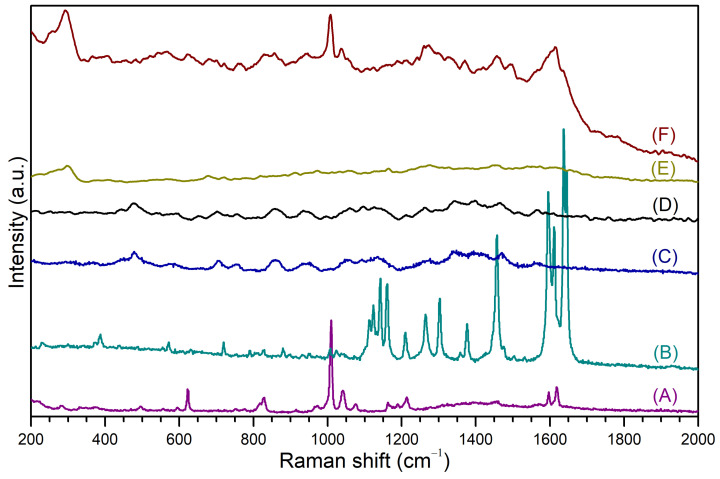
Raman spectra of (**A**) PhEA, (**B**) PIP, (**C**) CCD/P, and (**D**) CCD/P-PhEA-PIP; SERS spectra of (**E**) AuNS-CCD/P and (**F**) AuNS-CCD/P-PhEA-PIP.

Raman spectroscopy was used to study the co-loading of PhEA and PIP in the AuNS-CCD/P nanomaterial and to determine important interactions of these drugs with the gold atoms on the surface of the AuNS-CCD/P-PhEA-PIP system. Figure 9 shows the Raman spectra of (A) PhEA, (B) PIP, (C) CCD/P, and (D) CCD/P-PhEA-PIP and the SERS spectra of (E) AuNS-CCD/P and (F) AuNS-CCD/P-PhEA-PIP. Rigorous theoretical work was performed to assign molecular vibrations of the Raman and SERS spectra using two approaches, B3LYP and B3LYP/VPT2, to obtain full harmonic spectra and full anharmonic spectra, respectively. Then, the VPT2 approach was used to correct overestimated (by 4–5%) harmonic fundamental DFT frequency with respect to the experimental frequency values; furthermore, VPT2 can recover the first overtones and combination bands (C.B.) because not all signals assigned by the harmonic calculation correspond to fundamental vibrations in the experimental vibrational spectra (additional details are shown in the Supplementary Material, Sections S4 and S5).

The Raman spectra of PhEA (A) and PIP (B) were directly compared to the SERS spectrum of AuNS-CCD/P-PhEA-PIP (F). The CCD/P (C) and CCD/P-PhEA-PIP (D) systems showed similar Raman spectra; thus, it was not possible to assign or recognize the signals of both drugs. The registered signals and their assignments are shown in Table 3.

In the case of PhEA, the signal at 829 cm^−1^ corresponded to amine H wagging, which shifted to 827 cm^−1^ due to the interaction of the amino groups with the AuNSs surface. The other signals corresponded to the vibrations associated with the aryl rings, which were displaced due to the inclusion in the CCD/P. The vibrations at 1009, 1040, 1164, 1597, and 1617 cm^−1^ in the PhEA spectra were detected and were parallel to the aryl axis.

In the SERS spectrum, the two signals corresponding to the C=C symmetrical bending and to H rocking with deformation of the aryl ring were shifted to 1008 and 1036 cm^−1^, respectively, corresponding to the most intense signals. This result suggests that PhEA included in CCD/P was oriented perpendicular (┴) to the AuNSs surface probably due to the NH_2_–Au interactions.

The signals at 1111, 1237, 1302, and 1376 cm^−1^ were related to the H vibrations of the aryl and diene groups, and these signals were displaced in the SERS spectra due to the inclusion of PIP onto CCD/P.

The signals at 1122, 1141, 1595, 1610, 1636, and 1645 cm^−1^ in the PIP spectrum corresponded to the combined bands of piperidine, aryl and aliphatic vibrations. The signals at 1590, 1614, and 1637 cm^−1^ were the major peaks of PIP in the SERS spectra. These vibrations were not in a single plane, and changes in their intensity, disappearance or displacement were attributed to a parallel (‖) orientation to the surface of AuNS due to dynamic inclusion of PIP in CCD/P.

The SERS spectrum of the AuNS-CCD/P system had a signal at 293 cm^−1^, which was displaced to 297 cm^−1^ in the AuNS-CCD/P-PhEA-PIP spectrum. This signal corresponded to vibrations derived from the interaction between AuNSs and CCD/P.

**Figure 10:**
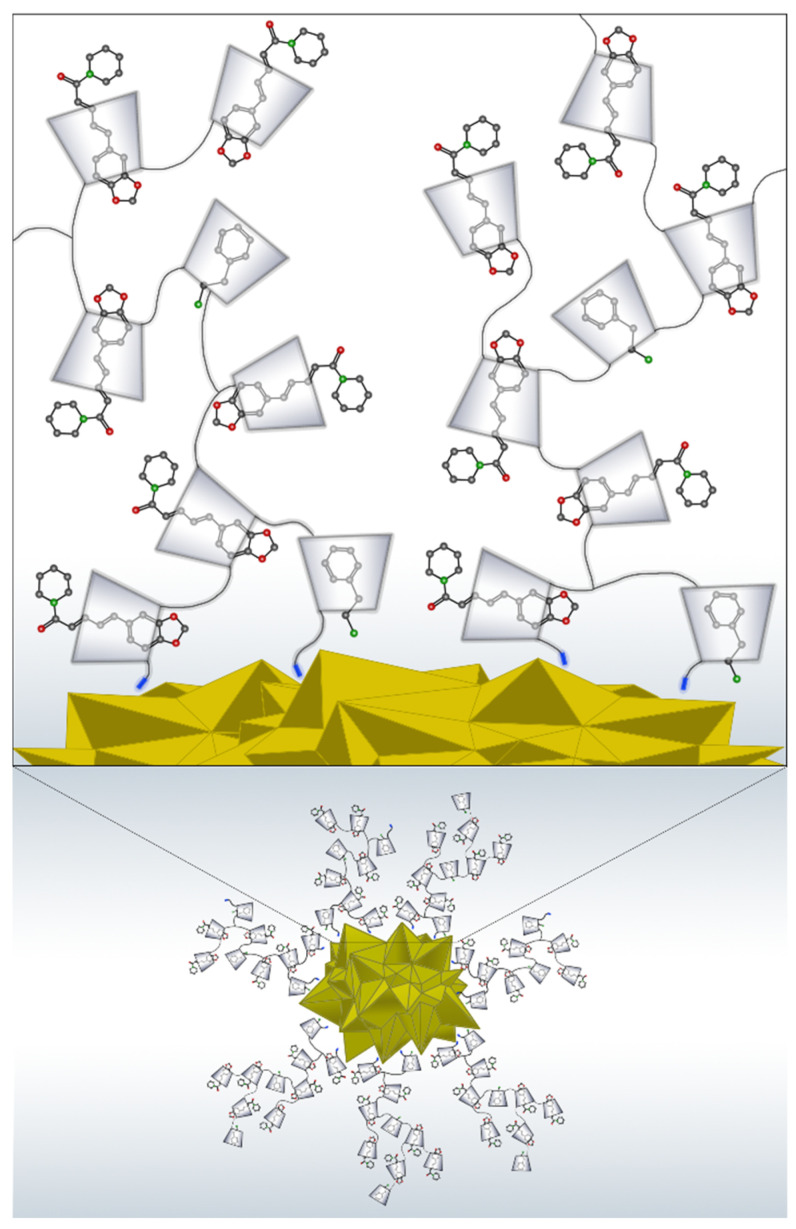
Schematic representation of the functionalization of AuNS with loaded CCD/P, forming the AuNS-CCD/P-PhEA-PIP nanosystem.

The differences in the SERS and Raman spectra, mainly in the relative intensity and wavenumber shifts, were related to different orientations of the analytes on the AuNSs surface according to SERS selection rules [107,108]. Therefore, the PhEA and PIP molecules were identified using SERS. Additionally, both drugs acquired a preferential orientation upon inclusion in the polymer and interaction with the surface atoms of AuNSs. A scheme of the AuNS-CCD/P-PhEA-PIP system based on the information provided by SERS and other techniques is shown in Figure 10.

Highly anisotropic nanoparticles, such as AuNSs, have high SERS enhancement factors [109,110,111]. Thus, the information obtained in this study was complemented by a rigorous theoretical analysis; this approach is effective for identification of molecules located near the surface of the gold atoms and may be used to evaluate their spatial orientation, which may be relevant for the construction of nanosystems for biomedical applications using SERS-based detection and/or imaging.

## 4. Conclusions

Minor adjustments to the reported method of AuNS synthesis increased colloidal stability of the particles over time. Spontaneous agglomeration and resuspension enabled functionalization and purification of AuNSs. CCD/P was used to functionalize AuNSs and effectively load the PhEA and PIP drugs to generate a new AuNS-CCD/P-PhEA-PIP nanomaterial. Loading of the drugs in the polymer was 95 ± 7%. The highly anisotropic surface of AuNSs enabled the investigation of the composition, interaction and orientation of both drugs in the nanosystem using SERS. Thus, this unique nanomaterial can be used for simultaneous drug loading and in the studies of SERS-based detection, leading to potential applications in therapeutic drug monitoring or theranostics.

## Figures and Tables

**Table 1:** Proton assignments and ^1^H-NMR chemical shifts of free phenylethylamine and piperine and compounds interacting with the cavities of cationic β-cyclodextrin-based polymer.

H of PhEA	δPhEA (ppm)	δCCD/P-PhEA-PIP (ppm)	Δδ (ppm)	H of PIP	δPIP (ppm)	δCCD/P-PhEA-PIP (ppm)	Δδ (ppm)
Hb	2.658	2.683	0.025	Ho	1.475	1.475	0
Ha	2.795	2.819	0.024	Hp	1.594	1.594	0
He	7.176	7.171	−0.005	Hn	3.521	-	-
Hc	7.205	7.198	−0.007	Hm	6.043	6.040	−0.003
Hd	7.285	7.278	−0.007	Hi	6.671	6.666	−0.005
				Hj	6.894	6.893	−0.001
				Hl	6.922	6.922	0
				Hk	6.961	6.960	−0.001
				Hf	6.990/6.850	6.990/6.850	0
				Hh/Hg	7.171/7.219	7.165/7.215	−0.006/−0.004

**Table 2:** Wavelengths of the absorbance maxima and plasmon bandwidth according to the UV–VIS spectra, hydrodynamic diameter, polydispersity index (PDI), surface charge, and diameter according to TEM for AuNSs, AuNS-CCD/P, and AuNS-CCD/P-PhEA-PIP.

System	Wavelength of Abs*_max_* (nm)	Plasmon Bandwidth (nm)	Hydrodynamic Diameter (nm)	PDI	Surface Charge (mV)	TEM Diameter (nm)
AuNSs	639	275	121 ± 18	0.220	−48.9 ± 3.1	83 ± 30
AuNS-CCD/P	610	245	167 ± 36	0.418	−15.9 ± 0.6	87 ± 27
AuNS-CCD/P-PhEA-PIP	618	246	178 ± 39	0.551	−15.5 ± 0.2	78 ± 23

**Table 3:** Experimental and theoretical Raman signals of PhEA and PIP, SERS bands of the AuNS-CCD/P-PhEA-PIP system and most likely band assignments. Combination bands (C.B.).

Phenylethylamine				Piperine			
Raman Signal (cm^−1^)	Theoretical Raman (cm^−1^)	SERS Signal (cm^−1^)	Assigned Molecular Vibration	Raman Signal (cm^−1^)	Theoretical Raman (cm^−1^)	SERS Signal (cm^−1^)	Assigned Molecular Vibration
622	629	624	-Symmetrical aryl ring deformation	1111	1085	1106	-Aryl ring H scissoring
829	810	827	-Amine H wagging	1122	1116	1124	- H of piperidine aliphatic chain twisting and H of methylene dioxy group twisting
1009	999	1008	-C=C aryl ring symmetrical bending	1141	1146	1146	- H of piperidine aliphatic chain twisting, C-C diene chain stretching and aryl ring H scissoring
1040	1036	1036	-Aryl ring H rocking and aryl deformation	1160	1150	1163	-C. B.: (i) Aryl ring out of plane torsion and diene chain out of plane torsion; (ii) aryl ring bending deformation
1164	1167	1163	-Aryl ring H scissoring	1214	1226	1210	- H of piperidine aliphatic chain twisting and H of diene chain rocking
1214	1207	1210	-C. B.: (i) Aryl ring out of plane torsion and deformation; (ii) aryl ring H wagging	1237	1277	1242	-Aryl ring H rocking
1597	1583	1602	-C=C aryl ring symmetrical stretching	1302	1312	1299	-Diene chain H scissoring
1617	1602	1614	-C=C aryl ring symmetrical stretching	1376	1360	1369	-Diene Chain H rocking
				1457	---	1459	------
				1595	1599	1590	-C. B.: (i) C-C-C piperidine aliphatic chain bending, C-C-N piperidine aliphatic chain-amide group bending and diene chain bending; (ii) piperidine aliphatic chain H twisting and diene chain H rocking
				1610	1606	1614	-C. B.: (i) Piperidine aliphatic chain H rocking, diene chain bending and aryl ring bending deformation; (ii) piperidine aliphatic chain H twisting, C-C diene chain stretching and aryl ring H scissoring
				1636	1635	1637	-C. B.: (i) C-C-C piperidine aliphatic chain bending; (ii) diene chain H rocking, aryl ring hydrogen scissoring and methylene dioxy group H twisting
				1645	1646	-	-C. B.: (i) Piperidine aliphatic chain H rocking and N-C(=O)-C amide group bending; (ii) piperidine aliphatic chain H twisting, C-C diene chain stretching and aryl ring H scissoring

## Data Availability

The data presented in this study are available on request from the corresponding author. The data are not publicly available due to privacy.

## Supplementary Materials

The following are available online at https://www.mdpi.com/1999-4923/13/2/261/s1, Section S1: Preparation and stabilization of gold nanostar; Section S2: Formation of cationic β-cyclodextrin-based polymers and drug loading; Section S3: Stability of gold nanostars with cationic β-cyclodextrin-based polymer and cationic β-cyclodextrin-based polymer including phenylethylamine and piperine; Section S4: Theoretical methods; Section S5: Comparison of theoretical estimation of Raman activity with experimental Raman spectra.

## References

[1] Tapia-Arellano A., Gallardo-Toledo E., Ortiz C., Henríquez J., Feijóo C.G., Araya E., Sierpe R., Kogan M.J. (2021). Functionalization with PEG/Angiopep-2 peptide to improve the delivery of gold nanoprisms to central nervous system: In vitro and in vivo studies. Mater. Sci. Eng. C.

[2] Yeh Y.C., Creran B., Rotello V.M. (2012). Gold nanoparticles: Preparation, properties, and applications in bionanotechnology. Nanoscale.

[3] Daniel M.C., Astruc D. (2004). Gold Nanoparticles: Assembly, Supramolecular Chemistry, Quantum-Size-Related Properties, and Applications toward Biology, Catalysis, and Nanotechnology. Chem. Rev..

[4] Kumar A., Mazinder Boruah B., Liang X.J. (2011). Gold nanoparticles: Promising nanomaterials for the diagnosis of cancer and HIV/AIDS. J. Nanomater..

[5] Shi J., Votruba A.R., Farokhzad O.C., Langer R. (2010). Nanotechnology in drug delivery and tissue engineering: From discovery to applications. Nano Lett..

[6] Guo J., Rahme K., He Y., Li L.L., Holmes J.D., O’Driscoll C.M. (2017). Gold nanoparticles enlighten the future of cancer theranostics. Int. J. Nanomed..

[7] Murphy C.J., Gole A.M., Stone J.W., Sisco P.N., Alkilany A.M., Goldsmith E.C., Baxter S.C. (2008). Gold nanoparticles in biology: Beyond toxicity to cellular imaging. Acc. Chem. Res..

[8] Jara-Guajardo P., Cabrera P., Celis F., Soler M., Berlanga I., Parra-Muñoz N., Acosta G., Albericio F., Guzman F., Campos M. (2020). Gold nanoparticles mediate improved detection of β-amyloid aggregates by fluorescence. Nanomaterials.

[9] Morales-Zavala F., Arriagada H., Hassan N., Velasco C., Riveros A., Álvarez A.R., Minniti A.N., Rojas-Silva X., Muñoz L.L., Vasquez R. (2017). Peptide multifunctionalized gold nanorods decrease toxicity of β-amyloid peptide in a Caenorhabditis elegans model of Alzheimer’s disease. Nanomed. Nanotechnol. Biol. Med..

[10] Hassan N., Cordero M.L., Sierpe R., Almada M., Juárez J., Valdez M., Riveros A., Vargas E., Abou-Hassan A., Ruso J.M. (2018). Peptide functionalized magneto-plasmonic nanoparticles obtained by microfluidics for inhibition of β-amyloid aggregation. J. Mater. Chem. B.

[11] Bao C., Conde J., Pan F., Li C., Zhang C., Tian F., Liang S., de la Fuente J.M., Cui D. (2016). Gold nanoprisms as a hybrid in vivo cancer theranostic platform for in situ photoacoustic imaging, angiography, and localized hyperthermia. Nano Res..

[12] Xu J.Y., Wang J., Kong L.T., Zheng G.C., Guo Z., Liu J.H. (2011). SERS detection of explosive agent by macrocyclic compound functionalized triangular gold nanoprisms. J. Raman Spectrosc..

[13] Wu X., Ming T., Wang X., Wang P., Wang J., Chen J. (2010). High-photoluminescence-yield gold nanocubes: For cell imaging and photothermal therapy. ACS Nano.

[14] Zhu J., Zhang F., Chen B.B., Li J.J., Zhao J.W. (2015). Tuning the shell thickness-dependent plasmonic absorption of Ag coated Au nanocubes: The effect of synthesis temperature. Mater. Sci. Eng. B Solid State Mater. Adv. Technol..

[15] Liu Y., Ashton J.R., Moding E.J., Yuan H., Register J.K., Fales A.M., Choi J., Whitley M.J., Zhao X., Qi Y. (2015). A plasmonic gold nanostar theranostic probe for in vivo tumor imaging and photothermal therapy. Theranostics.

[16] Chen H., Zhang X., Dai S., Ma Y., Cui S., Achilefu S., Gu Y. (2013). Multifunctional gold nanostar conjugates for tumor imaging and combined photothermal and chemo-therapy. Theranostics.

[17] Mousavi S.M., Zarei M., Hashemi S.A., Ramakrishna S., Chiang W.H., Lai C.W., Gholami A. (2020). Gold nanostars-diagnosis, bioimaging and biomedical applications. Drug Metab. Rev..

[18] del Valle A.C., Su C.-K., Sun Y.-C., Huang Y.-F. (2020). NIR-cleavable drug adducts of gold nanostars for overcoming multidrug-resistant tumors. Biomater. Sci..

[19] Liu X.L., Wang J.H., Liang S., Yang D.J., Nan F., Ding S.J., Zhou L., Hao Z.H., Wang Q.Q. (2014). Tuning plasmon resonance of gold nanostars for enhancements of nonlinear optical response and raman scattering. J. Phys. Chem. C.

[20] Barbosa S., Agrawal A., Rodríguez-Lorenzo L., Pastoriza-Santos I., Alvarez-Puebla R.A., Kornowski A., Weller H., Liz-Marzán L.M. (2010). Tuning size and sensing properties in colloidal gold nanostars. Langmuir.

[21] Yuan H., Khoury C.G., Hwang H., Wilson C.M., Grant G.A., Vo-Dinh T. (2012). Gold nanostars: Surfactant-free synthesis, 3D modelling, and two-photon photoluminescence imaging. Nanotechnology.

[22] Serrano-Montes A.B., Langer J., Henriksen-Lacey M., Jimenez De Aberasturi D., Solís D.M., Taboada J.M., Obelleiro F., Sentosun K., Bals S., Bekdemir A. (2016). Gold Nanostar-Coated Polystyrene Beads as Multifunctional Nanoprobes for SERS Bioimaging. J. Phys. Chem. C.

[23] Moram S.S.B., Byram C., Soma V.R. (2020). Gold-nanoparticle- and nanostar-loaded paper-based SERS substrates for sensing nanogram-level Picric acid with a portable Raman spectrometer. Bull. Mater. Sci..

[24] Meng X., Dyer J., Huo Y., Jiang C. (2020). Greater SERS Activity of Ligand-Stabilized Gold Nanostars with Sharp Branches. Langmuir.

[25] Theodorou I.G., Ruenraroengsak P., Gonzalez-Carter D.A., Jiang Q., Yagüe E., Aboagye E.O., Coombes R.C., Porter A.E., Ryan M.P., Xie F. (2019). Towards multiplexed near-infrared cellular imaging using gold nanostar arrays with tunable fluorescence enhancement. Nanoscale.

[26] Wang J., Zhou Z., Zhang F., Xu H., Chen W., Jiang T. (2018). A novel nanocomposite based on fluorescent turn-on gold nanostars for near-infrared photothermal therapy and self-theranostic caspase-3 imaging of glioblastoma tumor cell. Colloids Surf. B Biointerfaces.

[27] Wang Y., Serrano A.B., Sentosun K., Bals S., Liz-Marzán L.M. (2015). Stabilization and Encapsulation of Gold Nanostars Mediated by Dithiols. Small.

[28] Vega M.M., Bonifacio A., Lughi V., Marsi S., Carrato S., Sergo V. (2014). Long-term stability of surfactant-free gold nanostars. J. Nanopart. Res..

[29] Alinejad Z., Mahdavian A.R. (2018). Polymerization induced shape-tuning and multi-triggered switchability of gold nanostructures. Polymer.

[30] Borzenkov M., Chirico G., D’Alfonso L., Sironi L., Collini M., Cabrini E., Dacarro G., Milanese C., Pallavicini P., Taglietti A. (2015). Thermal and Chemical Stability of Thiol Bonding on Gold Nanostars. Langmuir.

[31] Loudy C.M., Chasvised S., Paybou C., Courrèges C., Allouche J., Martinez H., Bousquet A., Billon L. (2020). Revealing surface functionalities via microwave for the para-fluoro-Thiol click reaction. Polymer.

[32] Yuan H., Fales A.M., Vo-Dinh T. (2012). TAT Peptide-Functionalized Gold Nanostars: Enhanced Intracellular Delivery and Efficient NIR Photothermal Therapy Using Ultralow Irradiance. J. Am. Chem. Soc..

[33] Sasidharan S., Bahadur D., Srivastava R. (2017). Rapid, One-Pot, Protein-Mediated Green Synthesis of Gold Nanostars for Computed Tomographic Imaging and Photothermal Therapy of Cancer. ACS Sustain. Chem. Eng..

[34] Jana D., Matti C., He J., Sagle L. (2015). Capping Agent-Free Gold Nanostars Show Greatly Increased Versatility and Sensitivity for Biosensing. Anal. Chem..

[35] Rotz M.W., Culver K.S.B., Parigi G., Macrenaris K.W., Luchinat C., Odom T.W., Meade T.J. (2015). High relaxivity Gd(III)-DNA gold nanostars: Investigation of shape effects on proton relaxation. ACS Nano.

[36] Mariani S., Scarano S., Spadavecchia J., Minunni M. (2015). A reusable optical biosensor for the ultrasensitive and selective detection of unamplified human genomic DNA with gold nanostars. Biosens. Bioelectron..

[37] Liang S., Li C., Zhang C., Chen Y., Xu L., Bao C., Wang X., Liu G., Zhang F., Cui D. (2015). CD44v6 monoclonal antibody-conjugated gold nanostars for targeted photoacoustic imaging and plasmonic photothermal therapy of gastric cancer stem-like cells. Theranostics.

[38] Zhang Y., Wang X.P., Perner S., Bankfalvi A., Schlücker S. (2018). Effect of Antigen Retrieval Methods on Nonspecific Binding of Antibody-Metal Nanoparticle Conjugates on Formalin-Fixed Paraffin-Embedded Tissue. Anal. Chem..

[39] Liu Y., Zhi X., Yang M., Zhang J., Lin L., Zhao X., Hou W., Zhang C., Zhang Q., Pan F. (2017). Tumor-triggered drug release from calcium carbonate-encapsulated gold nanostars for near-infrared photodynamic/photothermal combination antitumor therapy. Theranostics.

[40] Tian F., Conde J., Bao C., Chen Y., Curtin J., Cui D. (2016). Gold nanostars for efficient in vitro and in vivo real-time SERS detection and drug delivery via plasmonic-tunable Raman/FTIR imaging. Biomaterials.

[41] Li Y., Zhai M., Xu H. (2019). Controllable synthesis of sea urchin-like gold nanoparticles and their optical characteristics. Appl. Surf. Sci..

[42] Wang H., Wu Y., Song H. (2019). Synergistic effects of photonic crystal and gold nanostars for quantitative SERS detection of 3-Phenoxybenzoic acid. Appl. Surf. Sci..

[43] Theodorou I.G., Jawad Z.A.R., Jiang Q., Aboagye E.O., Porter A.E., Ryan M.P., Xie F. (2017). Gold Nanostar Substrates for Metal-Enhanced Fluorescence through the First and Second Near-Infrared Windows. Chem. Mater..

[44] Haleem A., Chen J., Guo X.X., Wang J.Y., Li H.J., Li P.Y., Chen S.Q., He W.D. (2020). Hybrid cryogels composed of P(NIPAM-co-AMPS) and metal nanoparticles for rapid reduction of p-nitrophenol. Polymer.

[45] Tan B., Baycan F. (2020). A new donor-acceptor conjugated polymer-gold nanoparticles biocomposite materials for enzymatic determination of glucose. Polymer.

[46] Liu X., Liu F., Astruc D., Lin W., Gu H. (2019). Highly-branched amphiphilic organometallic dendronized diblock copolymer: ROMP synthesis, self-assembly and long-term Au and Ag nanoparticle stabilizer for high-efficiency catalysis. Polymer.

[47] Hernández Montoto A., Llopis-Lorente A., Gorbe M., Terrés J.M., Cao-Milán R., Díaz de Greñu B., Alfonso M., Ibañez J., Marcos M.D., Orzáez M. (2019). Janus Gold Nanostars–Mesoporous Silica Nanoparticles for NIR-Light-Triggered Drug Delivery. Chem. A Eur. J..

[48] Crini G. (2014). Review: A history of cyclodextrins. Chem. Rev..

[49] Szejtli J. (2004). Past, present, and future of cyclodextrin research. Pure Appl. Chem..

[50] Zhang J., Ma P.X. (2013). Cyclodextrin-based supramolecular systems for drug delivery: Recent progress and future perspective. Adv. Drug Deliv. Rev..

[51] Davis M.E., Brewster M.E. (2004). Cyclodextrin-based pharmaceutics: Past, present and future. Nat. Rev. Drug Discov..

[52] Silva N., Riveros A., Yutronic N., Lang E., Chornik B., Guerrero S., Samitier J., Jara P., Kogan M. (2018). Photothermally Controlled Methotrexate Release System Using β-Cyclodextrin and Gold Nanoparticles. Nanomaterials.

[53] Sierpe R., Noyong M., Simon U., Aguayo D., Huerta J., Kogan M.J., Yutronic N. (2017). Construction of 6-thioguanine and 6-mercaptopurine carriers based on βcyclodextrins and gold nanoparticles. Carbohydr. Polym..

[54] Asela I., Noyong M., Simon U., Andrades-Lagos J., Campanini-Salinas J., Vásquez-Velásquez D., Kogan M., Yutronic N., Sierpe R. (2017). Gold nanoparticles stabilized with βcyclodextrin-2-amino-4-(4-chlorophenyl) thiazole complex: A novel system for drug transport. PLoS ONE.

[55] Sierpe R., Lang E., Jara P., Guerrero A.R., Chornik B., Kogan M.J., Yutronic N. (2015). Gold Nanoparticles Interacting with β-Cyclodextrin-Phenylethylamine Inclusion Complex: A Ternary System for Photothermal Drug Release. ACS Appl. Mater. Interfaces.

[56] Osman S.K., Brandl F.P., Zayed G.M., Teßmar J.K., Göpferich A.M. (2011). Cyclodextrin based hydrogels: Inclusion complex formation and micellization of adamantane and cholesterol grafted polymers. Polymer.

[57] Kilsdonk E.P.C., Yancey P.G., Stoudt G.W., Bangerter F.W., Johnson W.J., Phillips M.C., Rothblat G.H. (1995). Cellular cholesterol efflux mediated by cyclodextrins. J. Biol. Chem..

[58] Li J., Xiao H., Li J., Zhong Y. (2004). Drug carrier systems based on water-soluble cationic β-cyclodextrin polymers. Int. J. Pharm..

[59] Herrera B.A., Bruna T.C., Sierpe R.A., Lang E.P., Urzúa M., Flores M.I., Jara P.S., Yutronic N.I. (2020). A surface functionalized with per-(6-amino-6-deoxy)-β-cyclodextrin for potential organic pollutant removal from water. Carbohydr. Polym..

[60] Yi W.J., Li L.J., He H., Hao Z., Liu B., Shen Y., Chao Z.S. (2018). Poly(L-lactide)/cyclodextrin/citrate networks modified hydroxyapatite and its role as filler in the promotion to the properties of poly(L-lactide) biomaterials. Polymer.

[61] Shao Y., Jia Y.G., Shi C., Luo J., Zhu X.X. (2014). Block and random copolymers bearing cholic acid and oligo(ethylene glycol) pendant groups: Aggregation, thermosensitivity, and drug loading. Biomacromolecules.

[62] Jiang Z., Liu H., He H., Ribbe A.E., Thayumanavan S. (2020). Blended Assemblies of Amphiphilic Random and Block Copolymers for Tunable Encapsulation and Release of Hydrophobic Guest Molecules. Macromolecules.

[63] Guo Q., Zhang T., An J., Wu Z., Zhao Y., Dai X., Zhang X., Li C. (2015). Block versus Random Amphiphilic Glycopolymer Nanopaticles as Glucose-Responsive Vehicles. Biomacromolecules.

[64] Malanga M., Szemán J., Fenyvesi É., Puskás I., Csabai K., Gyémánt G., Fenyvesi F., Szente L. (2016). “Back to the Future”: A New Look at Hydroxypropyl Beta-Cyclodextrins. J. Pharm. Sci..

[65] Kiss T., Fenyvesi F., Bácskay I., Váradi J., Fenyvesi É., Iványi R., Szente L., Tósaki Á., Vecsernyés M. (2010). Evaluation of the cytotoxicity of β-cyclodextrin derivatives: Evidence for the role of cholesterol extraction. Eur. J. Pharm. Sci..

[66] Furuya T., Koga T. (2017). Theoretical study of inclusion complex formation of cyclodextrin and single polymer chain. Polymer.

[67] Zhong N., Ohvo-Rekilä H., Ramstedt B., Slotte J.P., Bittman R. (2001). Selective removal of palmitic acid from Langmuir monolayers by complexation with new quaternary ammonium β-cyclodextrin derivatives. Langmuir.

[68] Chen H., Kou X., Yang Z., Ni W., Wang J. (2008). Shape- and size-dependent refractive index sensitivity of gold nanoparticles. Langmuir.

[69] Isaacs S.R., Cutler E.C., Park J.S., Lee T.R., Shon Y.S. (2005). Synthesis of tetraoctylammonium-protected gold nanoparticles with improved stability. Langmuir.

[70] George Thomas K., Zajicek J., Kamat P.V. (2002). Surface binding properties of tetraoctylammonium bromide-capped gold nanoparticles. Langmuir.

[71] Chen S., Liu Y., Wu G. (2005). Stabilized and size-tunable gold nanoparticles formed in a quaternary ammonium-based room-temperature ionic liquid under γ-irradiation. Nanotechnology.

[72] Astruc D., Lu F., Aranzaes J.R. (2005). Nanoparticles as recyclable catalysts: The frontier between homogeneous and heterogeneous catalysis. Angew. Chemie Int. Ed..

[73] Vivek J.P., Burgess I.J. (2012). Quaternary ammonium bromide surfactant adsorption on low-index surfaces of gold. 2. Au(100) and the role of crystallographic-dependent adsorption in the formation of anisotropic nanoparticles. Langmuir.

[74] Farren-Dai M., Awoonor-Williams E., Macneil C.S., Mahimwalla Z., Ghandi K. (2014). A novel gold nanoparticle stabilization and its muon chemistry. Chem. Phys. Lett..

[75] Vivek J.P., Burgess I.J. (2012). Quaternary ammonium bromide surfactant adsorption on low-index surfaces of gold. 1. Au(111). Langmuir.

[76] Wankar J., Kotla N.G., Gera S., Rasala S., Pandit A., Rochev Y.A. (2020). Recent Advances in Host–Guest Self-Assembled Cyclodextrin Carriers: Implications for Responsive Drug Delivery and Biomedical Engineering. Adv. Funct. Mater..

[77] Peng L., Liu S., Feng A., Yuan J. (2017). Polymeric Nanocarriers Based on Cyclodextrins for Drug Delivery: Host-Guest Interaction as Stimuli Responsive Linker. Mol. Pharm..

[78] Irsfeld M., Spadafore M., Prüß B.M. (2013). β-phenylethylamine, a small molecule with a large impact. Webmedcentral.

[79] Szabo A., Billett E., Turner J. (2001). Phenylethylamine, a possible link to the antidepressant effects of exercise?. Br. J. Sports Med..

[80] Lee S.A., Hong S.S., Han X.H., Hwang J.S., Oh G.J., Lee K.S., Lee M.K., Hwang B.Y., Ro J.S. (2005). Piperine from the Fruits of Piper longum with Inhibitory Effect on Monoamine Oxidase and Antidepressant-Like Activity. Chem. Pharm. Bull..

[81] Kulkarni S.K., Bhutani M.K., Bishnoi M. (2008). Antidepressant activity of curcumin: Involvement of serotonin and dopamine system. Psychopharmacology.

[82] Zarai Z., Boujelbene E., Ben Salem N., Gargouri Y., Sayari A. (2013). Antioxidant and antimicrobial activities of various solvent extracts, piperine and piperic acid from Piper nigrum. LWT Food Sci. Technol..

[83] Kumar V., Patil V., Apte A., Harale N., Patil P., Kulkarni S. (2015). Ultrasensitive Gold Nanostar-Polyaniline Composite for Ammonia Gas Sensing. Langmuir.

[84] Otto L., Budde K., Kastenmüller G., Kaul A., Völker U., Völzke H., Adamski J., Kühn J.P., Krumsiek J., Artati A. (2020). Associations between adipose tissue volume and small molecules in plasma and urine among asymptomatic subjects from the general population. Sci. Rep..

[85] Minati L., Benetti F., Chiappini A., Speranza G. (2014). One-step synthesis of star-shaped gold nanoparticles. Colloids Surfaces A Physicochem. Eng. Asp..

[86] Ezawa T., Inoue Y., Tunvichien S., Suzuki R., Kanamoto I. (2016). Changes in the Physicochemical Properties of Piperine/ β -Cyclodextrin due to the Formation of Inclusion Complexes. Int. J. Med. Chem..

[87] Frisch M.J., Trucks G.W., Schlegel H.B., Scuseria G.E., Robb M.A., Cheeseman J.R., Scalmani G., Barone V., Mennucci B., Petersson G.A. (2013). Gaussian 09, Revision D.01.

[88] Becke A.D. (1988). Density-functional exchange-energy approximation with correct asymptotic behavior. Phys. Rev. A.

[89] Lee C., Yang W., Parr R.G. (1988). Development of the Colle-Salvetti correlation-energy formula into a functional of the electron density. Phys. Rev. B.

[90] Becke A.D. (1993). Density-functional thermochemistry. III. The role of exact exchange. J. Chem. Phys..

[91] Stephens P.J., Devlin F.J., Chabalowski C.F., Frisch M.J. (1994). Ab Initio calculation of vibrational absorption and circular dichroism spectra using density functional force fields. J. Phys. Chem..

[92] Balan C., Pop L.C., Baia M. (2019). IR, Raman and SERS analysis of amikacin combined with DFT-based calculations. Spectrochim. Acta Part A Mol. Biomol. Spectrosc..

[93] Barone V. (2005). Anharmonic vibrational properties by a fully automated second-order perturbative approach. J. Chem. Phys..

[94] Kooij E.S., Ahmed W., Hellenthal C., Zandvliet H.J.W., Poelsema B. (2012). From nanorods to nanostars: Tuning the optical properties of gold nanoparticles. Colloids Surf. A Physicochem. Eng. Asp..

[95] De Puig H., Tam J.O., Yen C.W., Gehrke L., Hamad-Schifferli K. (2015). Extinction Coefficient of Gold Nanostars. J. Phys. Chem. C.

[96] Wu Q., Sun Y., Ma P., Zhang D., Li S., Wang X., Song D. (2016). Gold nanostar-enhanced surface plasmon resonance biosensor based on carboxyl-functionalized graphene oxide. Anal. Chim. Acta.

[97] Babij N.R., McCusker E.O., Whiteker G.T., Canturk B., Choy N., Creemer L.C., Amicis C.V.D., Hewlett N.M., Johnson P.L., Knobelsdorf J.A. (2016). NMR Chemical Shifts of Trace Impurities: Industrially Preferred Solvents Used in Process and Green Chemistry. Org. Process Res. Dev..

[98] Ouellette R.J., Rawn J.D. (2014). Organic Chemistry-Structure, Mechanism, and Synthesis. Organic Chemistry.

[99] Ezawa T., Inoue Y., Murata I., Takao K., Sugita Y., Kanamoto I. (2018). Characterization of the Dissolution Behavior of Piperine/Cyclodextrins Inclusion Complexes. AAPS PharmSciTech.

[100] Ternes W., Krause E.L. (2002). Characterization and determination of piperine and piperine isomers in eggs. Anal. Bioanal. Chem..

[101] Abou-Okeil A., Rehan M., El-Sawy S.M., El-bisi M.K., Ahmed-Farid O.A., Abdel-Mohdy F.A. (2018). Lidocaine/β-cyclodextrin inclusion complex as drug delivery system. Eur. Polym. J..

[102] Abarca R.L., Rodríguez F.J., Guarda A., Galotto M.J., Bruna J.E. (2016). Characterization of beta-cyclodextrin inclusion complexes containing an essential oil component. Food Chem..

[103] Aytac Z., Kusku S.I., Durgun E., Uyar T. (2016). Encapsulation of gallic acid/cyclodextrin inclusion complex in electrospun polylactic acid nanofibers: Release behavior and antioxidant activity of gallic acid. Mater. Sci. Eng. C.

[104] Zhao X., Xiao D., Alonso J.P., Wang D.Y. (2017). Inclusion complex between beta-cyclodextrin and phenylphosphonicdiamide as novel bio-based flame retardant to epoxy: Inclusion behavior, characterization and flammability. Mater. Des..

[105] Dykman L., Khlebtsov N. (2012). Gold nanoparticles in biomedical applications: Recent advances and perspectives. Chem. Soc. Rev..

[106] Chhour P., Naha P.C., Cheheltani R., Benardo B., Mian S., Cormode D.P. (2016). Gold nanoparticles for biomedical applications: Synthesis and in vitro evaluation. Methods in Pharmacology and Toxicology.

[107] Moskovits M. (1985). Surface-enhanced spectroscopy. Rev. Mod. Phys..

[108] Aroca R. (2007). Surface-Enhanced Vibrational Spectroscopy.

[109] He S., Kang M.W.C., Khan F.J., Tan E.K.M., Reyes M.A., Kah J.C.Y. (2015). Optimizing gold nanostars as a colloid-based surface-enhanced Raman scattering (SERS) substrate. J. Opt..

[110] Nalbant Esenturk E., Hight Walker A.R. (2009). Surface-enhanced Raman scattering spectroscopy via gold nanostars. J. Raman Spectrosc..

[111] Abalde-Cela S., Aldeanueva-Potel P., Mateo-Mateo C., Rodríguez-Lorenzo L., Alvarez-Puebla R.A., Liz-Marzán L.M. (2010). Surface-enhanced Raman scattering biomedical applications of plasmonic colloidal particles. J. R. Soc. Interface.

