# Supplementary material for: Functionalization of Gold Nanostars with Cationic β-Cyclodextrin-Based Polymer for Drug Co-Loading and SERS Monitoring
Source: Pharmaceutics. 2021 Feb 15;13(2):261. doi: 10.3390/pharmaceutics13020261 (PMC7919026; doi:10.3390/pharmaceutics13020261)
Supplement: Supplementary file 1 [file pharmaceutics-13-00261-s001.pdf]

pharmaceutics

Supplementary Materials: Functionalization of Gold Nanostars with Cationic β-Cyclodextrin-Based Polymer for Drug Co-Loading and SERS Monitoring

Orlando Donoso-González [1,2,3], Lucas Lodeiro [4], Álvaro E. Aliaga [5], Miguel A. Laguna-Bercero [6], Soledad Bollo [3,7], Marcelo J. Kogan [2,3], Nicolás Yutronic [1,*], and Rodrigo Sierpe [1,2,3,7,*]

S1. Preparation and stabilization of gold nanostars

The tested dilution factors ranged from 2x to 10x; however, 3x to 10x dilutions resulted in stable hydrodynamic diameters of AuNSs in solution. Table S1 shows the hydrodynamic diameters of AuNSs in aqueous solution at various dilutions after zero, one, two, three and seven days. Figure S1 A shows the UV-VIS spectra of AuNSs at various dilutions, and Figure S1 B shows the UV-VIS spectra of AuNSs with a 3× dilution factor; the AuNSs were agglomerated 8 h after synthesis and were subsequently resuspended after eight hours and one, two and three days.

Table S1. Hydrodynamic diameter ±standard deviation of AuNSs in aqueous solution at various dilution factors (from 1x to 10x) after zero, one, two, three and seven days.

Dilution Factor	Hydrodynamic diameter (nm)				
	Day 0	Day 1	Day 2	Day 3	Day 7
1×	121 ± 23	657 ± 213	680 ± 242	725 ± 290	5560 ± 3205
2×	115 ± 20	598 ± 302	650 ± 283	666 ± 315	3578 ± 1780
3×	121 ± 18	137 ± 25	126 ± 24	132 ± 25	133 ± 25
4×	136 ± 22	134 ± 25	132 ± 31	140 ± 21	139 ± 28
5×	107 ± 15	111 ± 17	109 ± 21	120 ± 18	130 ± 24
6×	123 ± 20	132 ± 23	127 ± 25	135 ± 25	139 ± 30
7×	134 ± 32	136 ± 27	133 ± 28	122 ± 25	142 ± 33
8×	143 ± 33	148 ± 38	152 ± 36	155 ± 40	168 ± 43
9×	124 ± 34	107 ± 30	115 ± 31	123 ± 28	127 ± 30
10×	113 ± 30	108 ± 27	115 ± 25	118 ± 20	125 ± 26

Publisher's Note: MDPI stays neutral with regard to jurisdictional claims in published maps and institutional affiliations.

Copyright: © 2020 by the authors. Submitted for possible open access publication under the terms and conditions of the Creative Commons Attribution (CC BY) license (http://creativecommons.org/licenses/by/4.0/).

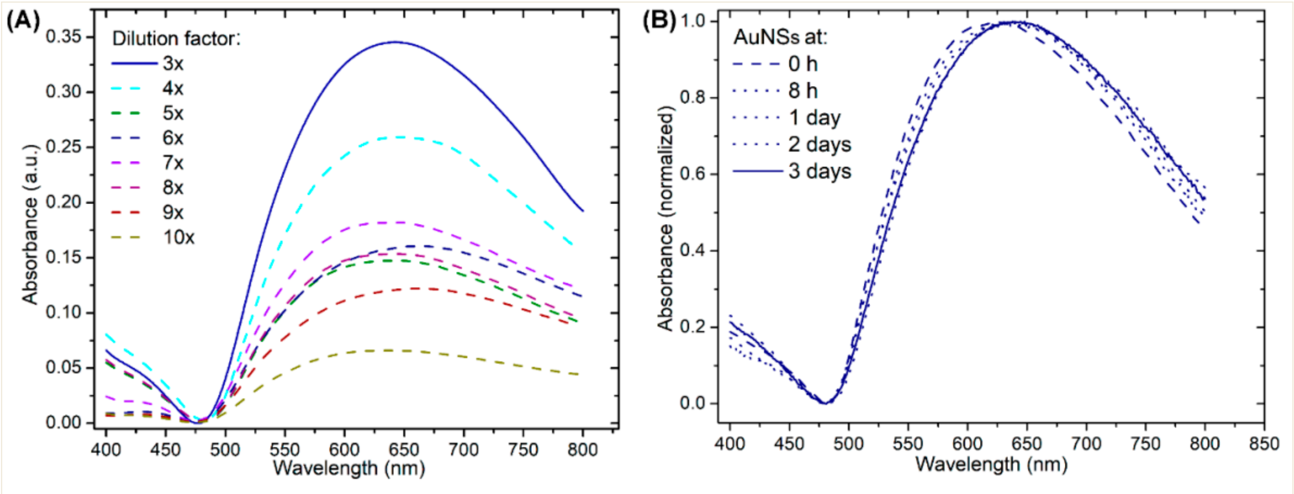

Figure S1. (A) Absorbance spectra of AuNSs with dilution factors between 3x and 10x. (B) Absorbance spectra of AuNSs with a 3x dilution factor; AuNSs were agglomerated eight hours after the synthesis and subsequently resuspended after eight hours and one, two and three days.

S2. Formation of cationic β-cyclodextrin-based polymers and drug loading

S2.1. Characterization of cationic β-cyclodextrin-based polymers using IR and mass spectrometry

The formation of CCD/P was characterized using IR spectroscopy. Figure S2 shows the IR spectra of (A) βCD, (B) CC, (C) EP and (D) CCD/P.

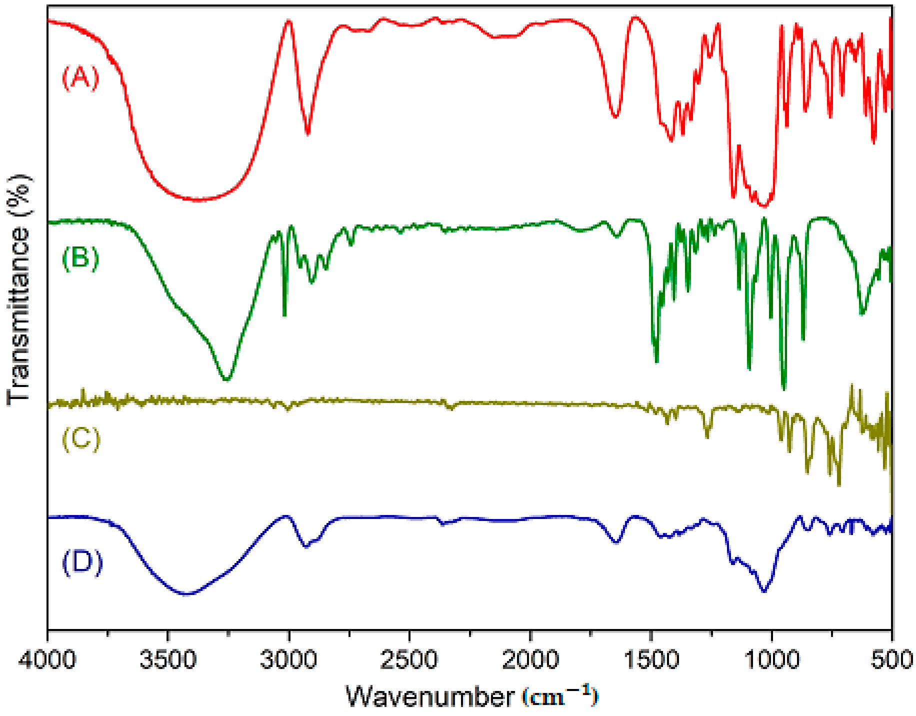

Figure S2. IR spectra of (A) β-cyclodextrin, (B) choline chloride, (C) epichlorohydrin and (D) cationic β-cyclodextrin-based polymers from 500 to 4000 cm^{-1}.

Figure S3 A and B show the mass spectra of CCD/P. mMass version 5.5.0 was used for the analyses [1–3]. The MALDI-TOF Proteins 5-20 kDa algorithm was used for spectral signal detection (m/z) with default parameters (signal-to-noise ratio of 2.5 and relative intensity limit of 1%). The maximum degree of polymerization corresponded to 14 units of βCD (m/z: 20,404.8429).

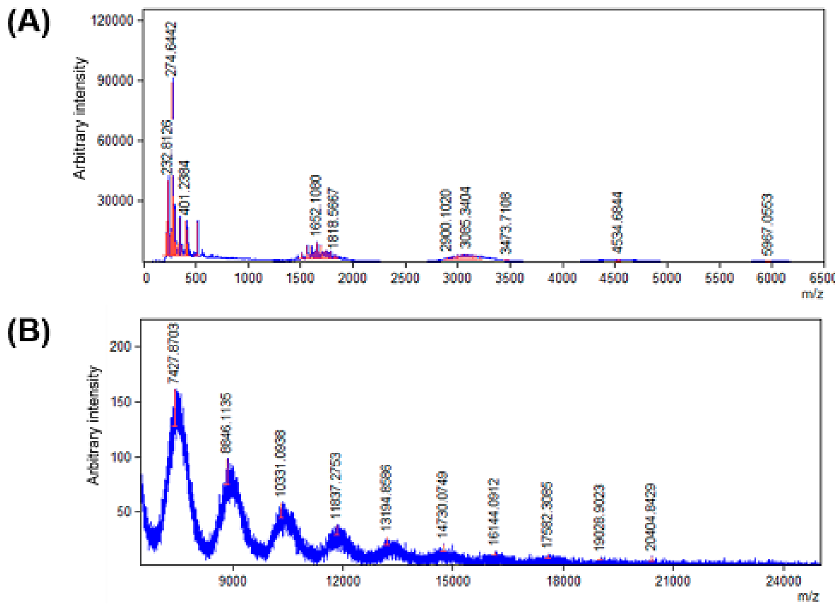

Figure S3. Mass spectra of CCD/P: (A) from 0 to 6,500 m/z and (B) from 7,000 to 25,000 m/z.

S2.2. Stoichiometric relationships between piperine, phenylethylamine and β-cyclodextrin

The stoichiometric ratio was calculated by comparing the integrals of the PhEA and PIP protons in the ^{1}H-NMR spectra of the CCD/P-PhEA-PIP system that included both drugs. The integrals of the PhEA signals (protons Ha, Hb and Hd) were assessed using the Hm, Ho and Hp signals of PIP integrated for 2, 4 and 2 protons, respectively, as a reference. Then, the integrals of the PIP signals (Hm, Ho and Hp) were assessed using the Ha, Hb and Hd signals of PhEA as a reference, and the values were integrated for 2 protons in each case (see the data of Tables S2 and S3).

Table S2. The values of integrated PhEA proton signals in the ^{1}H-NMR spectra of the CCD/P-PhEA-PIP system based on the integrated PIP proton signals used as a reference.

**Integration of PhEA protons**			**Integration of PIP protons**
Ha	Hb	Hd	(reference used)
0.66	0.75	0.60	Hm/∫ = 2
0.65	0.73	0.58	Ho/∫ = 4
0.58	0.66	0.52	Hp/∫ = 2

Table S3. The values of the integrated PIP proton signals in the ^{1}H-NMR spectra of the CCD/P-PhEA-PIP system based on the integrated PhEA proton signals used as a reference.

**Integration of PIP protons**			**Integration of PhEA protons**
Hm	Ho	Hp	(reference used)
6.76	13.89	7.71	Hd/∫ = 2
6.03	12.39	6.88	Ha/∫ = 2
5.03	10.89	6.04	Hb/∫ = 2

The average integrated PhEA proton signal was 0.64 (± 0.08) if the integrated PIP proton signal was 2; therefore, the stoichiometric ratio of PhEA to PIP was 1:3.

The stoichiometric ratio of CCD/P:drugs was evaluated by comparing the integrals of proton signals in the ^{1}H-NMR spectra of the CCD/P-PhEA-PIP system. The integrals of the Hm, Ho and Hp protons of PIP and the H1 protons of βCD were analyzed using the Ha, Hb and Hd signal protons of PhEA as reference, which integrated for 2 (see data in Table S4).

Table S4. The values of the integrated PIP and βCD proton signals in the ^{1}H-NMR spectra of the CCD/P-PhEA-PIP system based on the integrated PhEA proton signals used as a reference.

Integration of PIP protons			Integration of βCD proton	Integration of PhEA protons
Hm	Ho	Hp	H1	(reference used)
6.03	11.82	6.03	23.83	Hd/∫ = 2
6.03	11.82	6.03	23.31	Ha/∫ = 2
6.03	11.82	6.03	23.42	Hb/∫ = 2

The average integrated of the H1 protons of βCD was 23.5 (± 0.3) if the integrated PhEA proton signal was 2; therefore, the stoichiometric ratio of βCD:PhEA:PIP in the CCD/P system was 1:3:4 and the molar ratio CCD/P:drugs was 1:1.

S2.3. Characterization of cationic β-cyclodextrin-based polymers loaded with phenylethylamine and piperine using IR spectroscopy and TGA

The formation of the CCD/P-PhEA-PIP system was characterized using IR spectroscopy, and the spectrum was compared with the spectra of PhEA, PIP and CCD/P IR (see Figure S4).

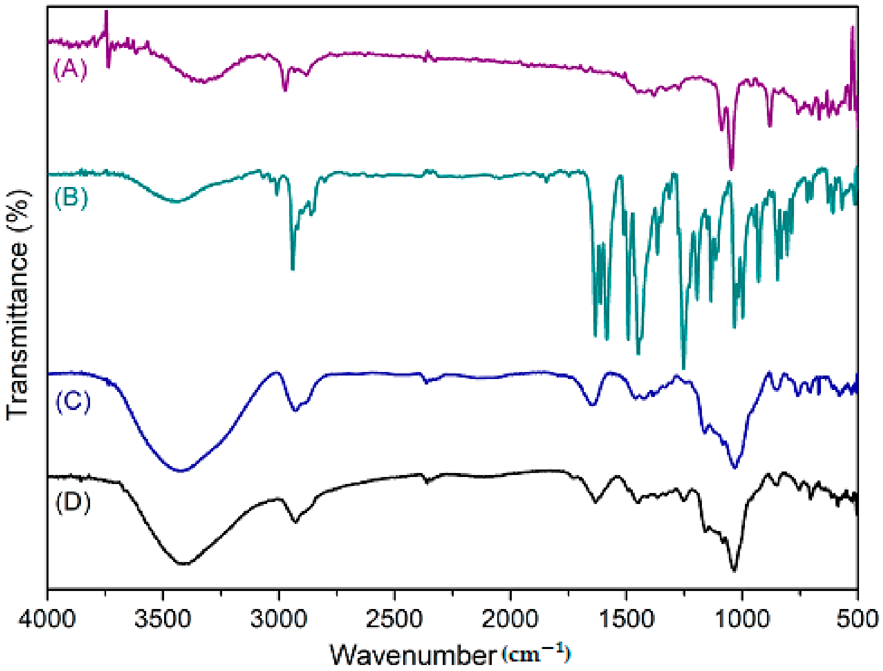

Figure S4. IR spectra of (A) PhEA, (B) PIP, (C) CCD/P and (D) CCD/P-PhEA-PIP.

Derivative curves were plotted to analyse the differences in the peaks of the thermogravimetric decomposition curves (Figure. 6 in the Results and Discussion, section 3.2).

Figure S5 shows the TGA derivative curve profiles of βCD (A), CCD/P (B) and CCD/P-PhEA-PIP (C).

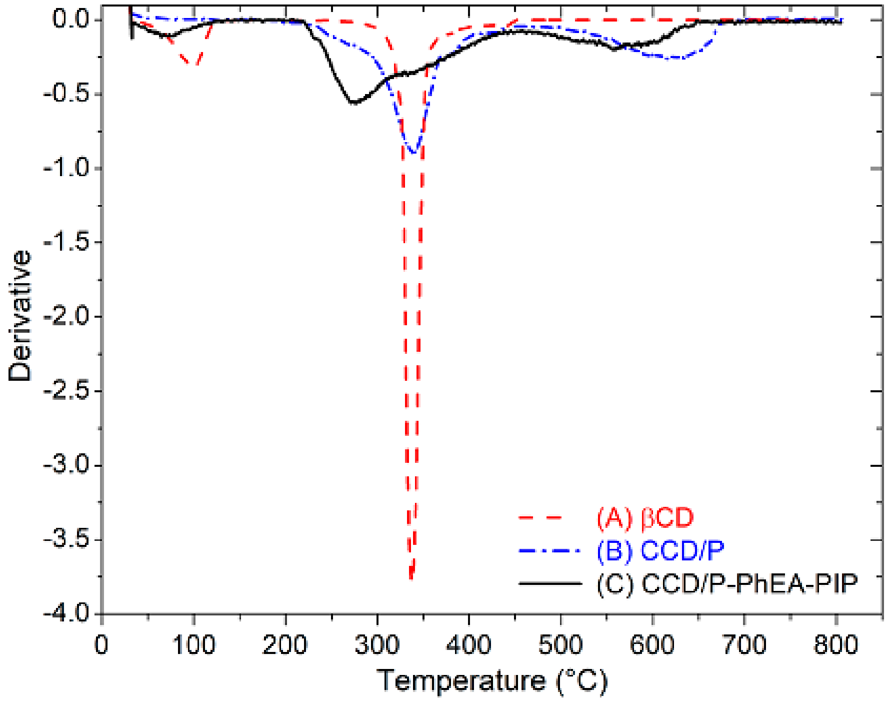

Figure S5. TGA derivative curves of (A) βCD, (B) CCD/P and (C) CCD/P-PhEA-PIP.

S2.4. Study of entrapment efficiency and loading capacity of cationic β-cyclodextrin-based polymers with phenylethylamine and piperine using the SPR technique

A gold foil was modified with an aqueous solution of 5.0 mM CCD/P to evaluate the entrapment efficiency and the loading capacity of the drugs at different concentrations. The drug solutions were prepared using ethanol in water (40% v/v), maintaining the 1:1 molar ratio of PhEA-PIP.

The SPR setup was done withtwo channels: the working and reference channels. The association curves were corrected by double reference, discounting the response obtained from the reference channel and an injection of a 40% *v*/*v* ethanol solution. After each assay, the gold surface with CCD/P was regenerated using DMSO (1 M) for 7 min, at a flow rate of 20 µL/min.

Figure S6A shows a sensorgram with the response versus time curves at different initial concentrations of PhEA-PIP. Figure S6B shows the calibration curve obtained. A linear relationship between the responses and the corresponding concentration of drug was observed with an R^2 of 0.9939.

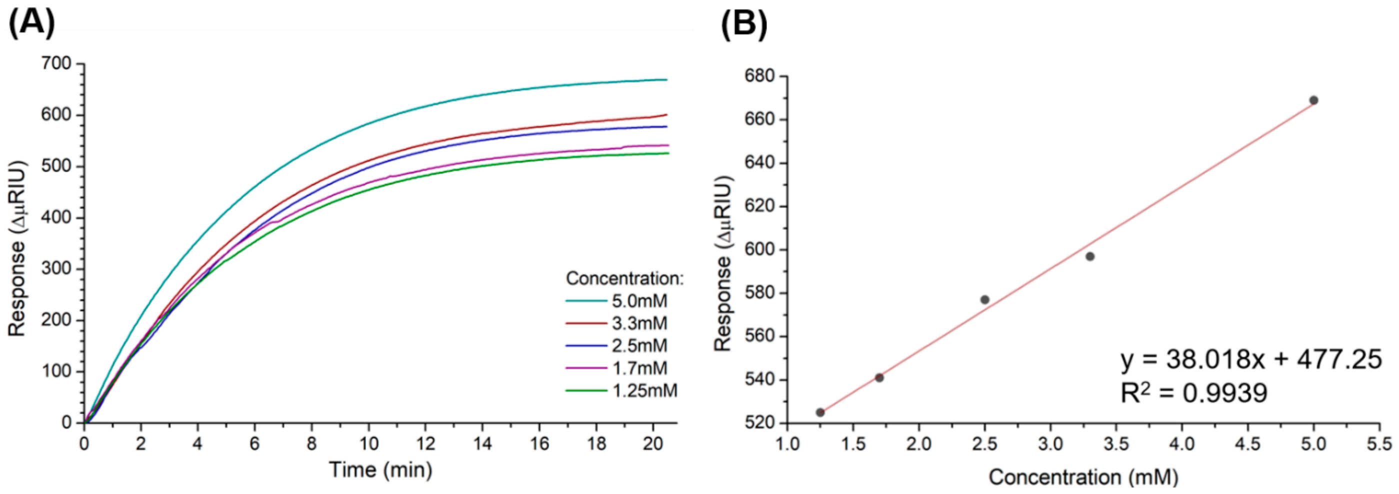

Figure S6. (A) Responses obtained with different initial concentrations of PhEA-PIP, and (B) Calibration curve of drug response versus total concentration, evaluated from 1.25 to 5.0 mM.

The data of the responses, concentrations, entrapment efficiency and loading capacity are summarized in Table S5. Each assay was performed in triplicate.

Table S5. Data obtained from PhEA-PIP responses, drug concentrations and percentages of entrapment efficiency and loading capacity.

**PhEA-PIP response (RIU)**	**Initial concentration (mM)**	**PhEA-PIP response included (ΔμRIU)**	**Calculated concentration (mM)**	**Entrapment efficiency (%)**	**Loading capacity (%)**
525	1.25	520 ± 6	1.1 ± 0.2	91 ± 16	28 ± 5
541	1.7	533 ± 2	1.5 ± 0.1	87 ± 3	37 ± 1
577	2.5	552 ± 12	2.0 ± 0.5	79 ± 18	49 ± 11
597	3.3	590 ± 11	3.0 ± 0.3	90 ± 12	74 ± 10
669	5.0	622 ± 8	3.8 ± 0.3	76 ± 6	95 ± 7

S3. Stability of gold nanostars with cationic β-cyclodextrin-based polymer and cationic β-cyclodextrin-based polymer including phenylethylamine and piperine

S3.1. Complementary characterization of gold nanostars with cationic β-cyclodextrin-based polymer

A study using the SPR technique was carried out to demonstrate the interaction between the quaternary ammonium groups of the polymer and the gold atoms of the AuNSs. Additionally, the degree of immobilization and the area of binding sites for the polymers onto a gold surface were calculated.

First, three successive injections of an aqueous solution of 5.0 mM CCD/P on a 50 nm thick gold foil were performed to reach saturation of its surface (see Figure S7A). Table S6 summarizes the data of responses and immobilization percentages for each injection (n=2). Then, four different gold foils were modified with a single injection of 5.0 mM CCD/P (see

Figure S7B). The corresponding responses and the average calculated are shown in Table S7 (n=4). The injection time was 30 min, at a flow rate of 5 μL/min.

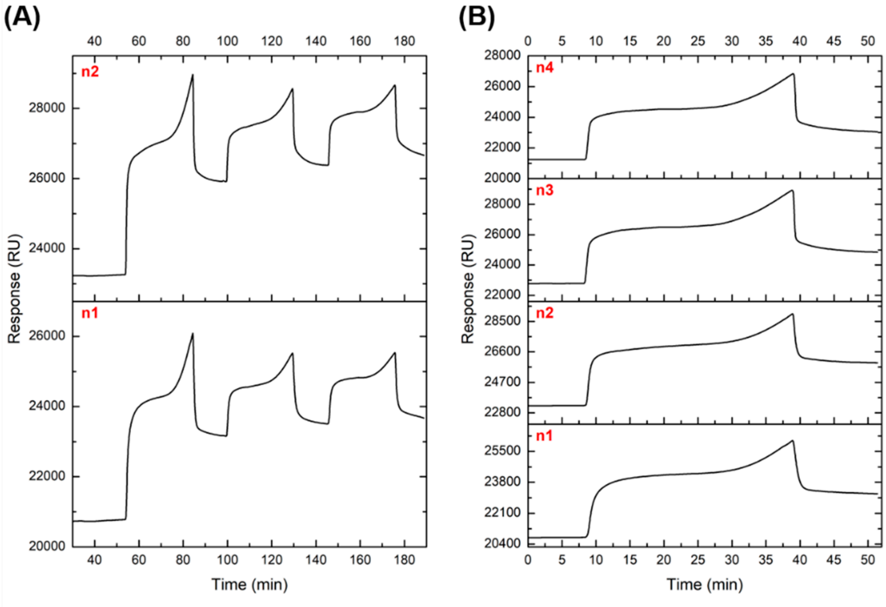

Figure S7. Response sensorgrams of: (A) 3 consecutive injections of 5.0 mM CCD/P for 30 minutes each (n=2) and (B) 1 injection of 5.0 mM CCD/P for 30 minutes (n=4).

Table S6. Average responses for the three consecutive injections of 5.0 mM CCD/P and degree of immobilization.

Injection number	Average response (ΔRU)	Degree of immobilization (%)
1	2549 ± 192	80 ± 6
2	390 ± 88	12 ± 3
3	234 ± 86	7 ± 3
total	3172 ± 183	100 ± 6

Table S7. Initial and final response, and response variation calculated for four gold surfaces modified with a 5.0 mM CCD/P injection.

Assay number	Initial response (RU)	Final response (RU)	Response variation (ΔRU)
1	20776	23189	2413
2	23258	25934	2676
3	22788	24867	2079

4	21242	23224	1982
Average	-	-	2288 (± 318)

Surface density and binding sites were obtained using the equations 1 and 2 shown below. The average response (2288 ± 318 RU) obtained for a single injection of 5.0 mM CCD/P was used in the first equation. The molecular weight of βCD used in the second equation was 1134.98 g/mol.

$$\text{Surface density} = \frac{\text{Response}_{\text{CCD/P}}}{1\text{x}10^{10}}\ (\text{g} \cdot \text{cm}^{-2}) \quad (1)$$

$$\text{Binding site} = \frac{\text{Surface density}}{\text{Mw}_{\beta-\text{Cyclodextrin}}}\ (\text{mol} \cdot \text{cm}^{-2}) \quad (2)$$

The first injection corresponded to 80% of the total coating, with a surface density of 2.3×10^{-7} ($\pm 3\text{x}10^{-8}$) g/cm^2 and an area of binding sites of $2.0\text{x}10^{-10}$ ($\pm 3\text{x}10^{-11}$) mol/cm^2. This demonstrates an efficient and nonsaturated immobilization of CCD/P on the gold surface, which could produce a higher degree of recognition of the drugs included into the βCD cavities. The entrapment efficiency and loading capacity studies were performed under these conditions and considered that the hydrophobic cavities of βCD were the only available active sites to include PhEA and PIP.

Once the interaction between the quaternary ammonium groups and the gold atoms was confirmed, we continued with the characterization of the AuNSs stabilized with CCD/P. Figure S8 shows the absorbance spectra of AuNSs and AuNS-CCD/P in aqueous solution obtained by UV-VIS spectroscopy. Figure S9 shows the ^{1}H-NMR spectra of (A) CCD/P and (B) CCD/P-stabilized AuNSs.

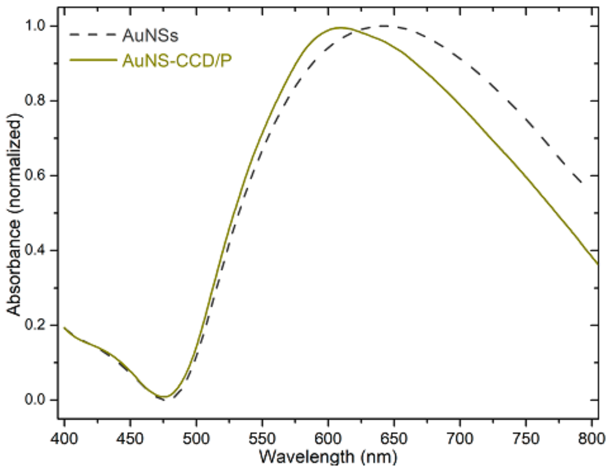

Figure S8. Absorbance spectra of AuNSs and AuNS-CCD/P.

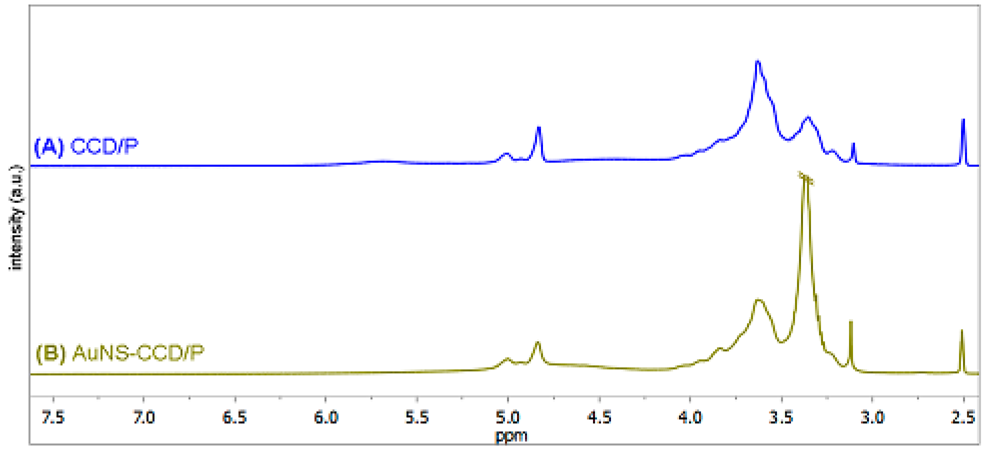

Figure S9. ^{1}H-NMR spectra of (A) CCD/P and (B) AuNS-CCD/P.

S3.2. Characterization of the stability of gold nanostars with cationic β-cyclodextrin-based polymer including phenylethylamine and piperine by UV-VIS spectroscopy and ^{1}H-NMR

Figure S10 shows the absorbance spectra of AuNSs and AuNSs stabilized with CCD/P-PhEA-PIP in aqueous solution. Figure S11shows the ^{1}H-NMR spectra of (A) CCD/P-PhEA-PIP and (B) AuNSs with CCD/P-PhEA-PIP.

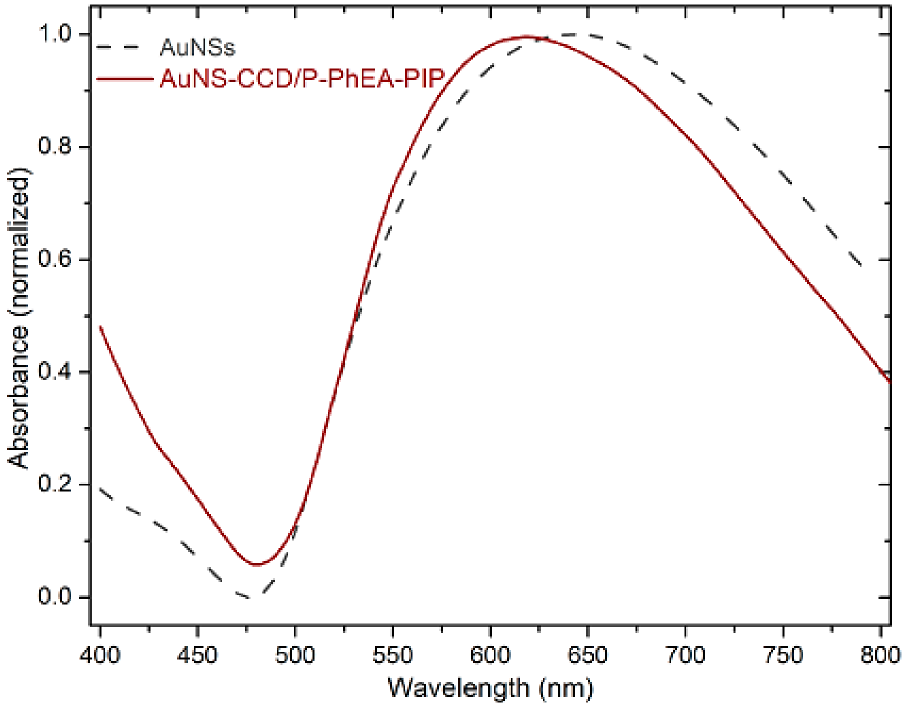

Figure S10. Absorbance spectra of AuNSs and AuNS-CCD/P-PhEA-PIP.

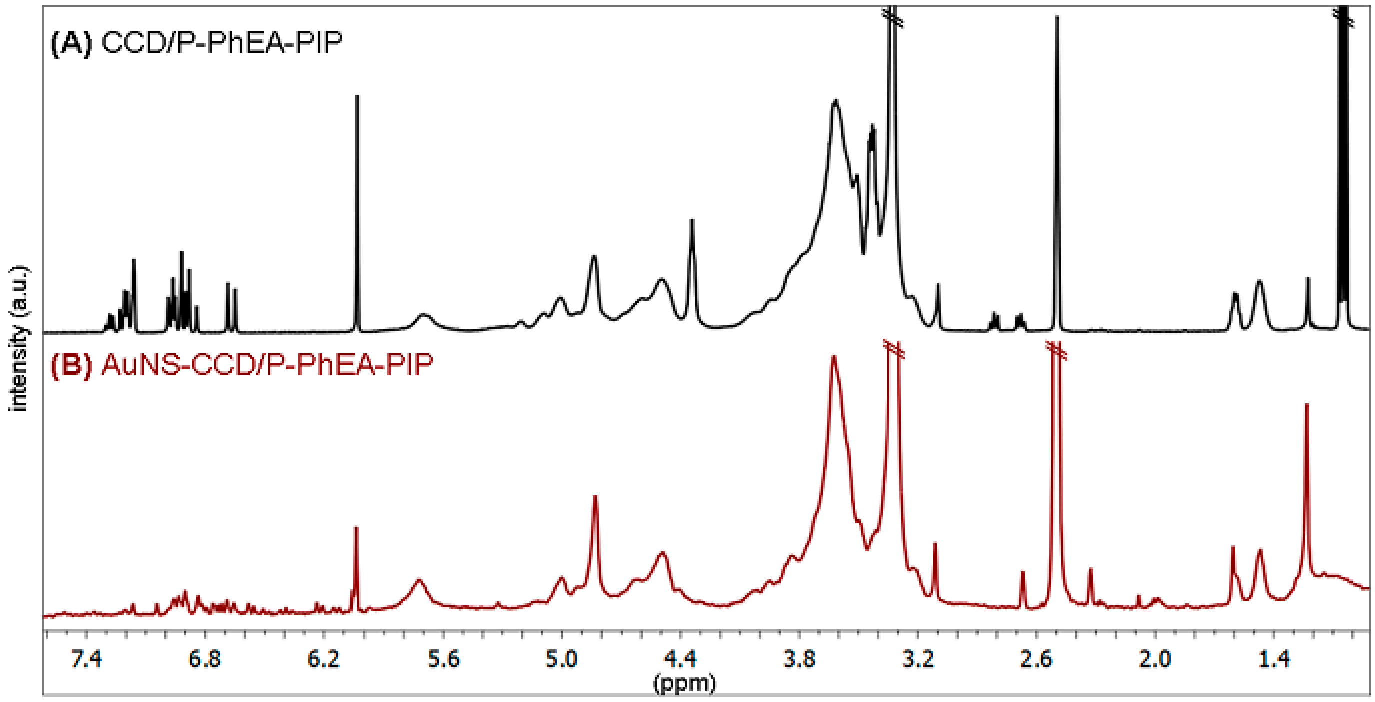

Figure S11. ^{1}H-NMR spectra of (A) CCD/P-PhEA-PIP and (B) AuNS-CCD/P-PhEA-PIP.

S3.3 Histograms of nanoparticle diameters obtained from TEM images

Figure S12 shows size distribution histograms for (A) AuNSs, (B) AuNS-CCD/P and (C) AuNS-CCD/P-PhEA-PIP from the TEM images shown in Figure 7 in the Results and Discussion, section 3.3. The diameters were 83 (± 30), 87 (± 27) and 78 (± 23) nm, respectively.

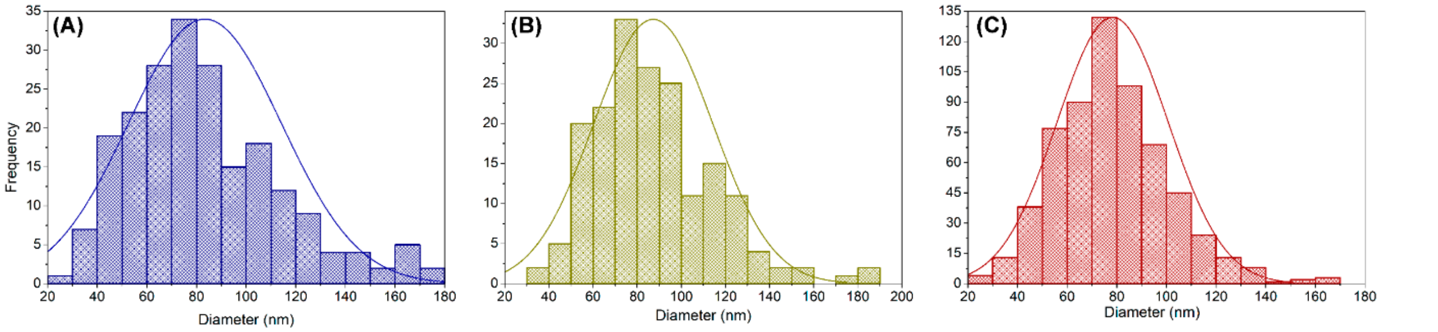

Figure S12. Size distribution histograms of (A) AuNSs, (B) AuNS-CCD/P and (C) AuNS-CCD/P-PhEA-PIP from TEM images.

S4. Theoretical methods

The anharmonic (VPT2) frequencies were assessed to optimize the assignment of the signals by two relevant criteria. First, the overestimation (4–5%) of harmonic fundamental DFT frequencies with respect to experimental frequency values was considered. These overestimations are due to various sources of error, such as basis set incompleteness, the exchange-correlation functional and mainly anharmonic corrections. Therefore, scale factors are frequently used to correct these values (in particular, a 0.96–0.97 scale factor is used for B3LYP) [4–6]. Comparison of calculated harmonic and anharmonic fundamental frequencies enabled to estimate the scale factors between the frequencies (0.961 and 0.963 for PhEA and PIP, respectively) in accordance with the reported scale factors (see Figure S13 A and B). The used functional and basis sets demonstrated optimal performance for

fundamental vibrational values, similar some benchmarks that compared the DFT functionals with MP2 [7] and by coupled cluster [6–8] calculations (post-Hartree-Fock Hamiltonians); however, the approach used in the present study had lower computational requirements.

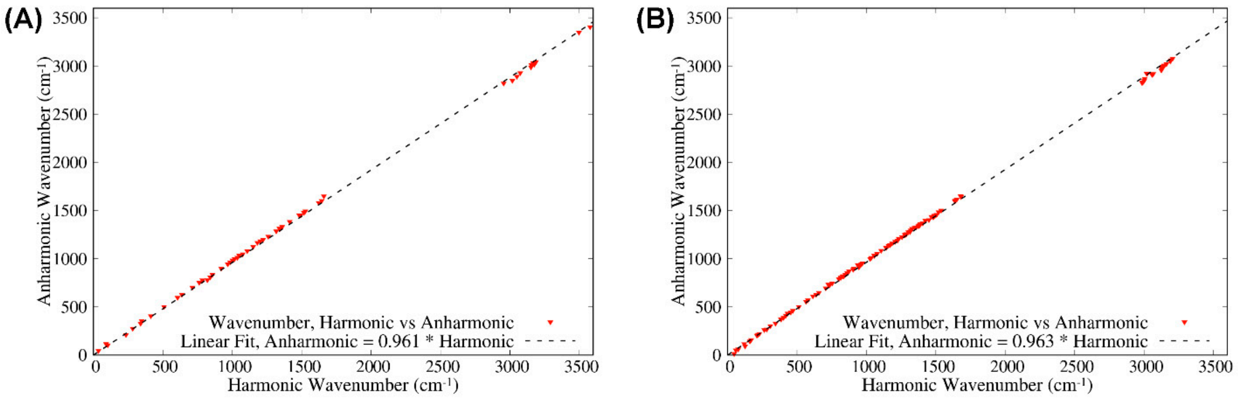

Figure S13. Comparison of fundamental anharmonic (VPT2) and harmonic wavenumber for all normal modes for (A) PhEA with a scale factor of 0.961 determined by linear fitting and for (B) PIP with a scale factor of 0.963 determined by linear fitting.

However, not all signals correspond to fundamental vibrations in experimental vibrational spectroscopy. Some signals are overtones, combination bands and Fermi resonances. The VPT2 approach can recover first overtones and combination bands beyond the anharmonic corrections for fundamental modes. Notably, the VPT2 approach requires numerical differentiation, preventing its practical use for medium and large molecules (similar to the present study) in conjunction with the post-Hartree-Fock methods. Other forms can be used to recover anharmonicities, overtones, combination bands and explicit solvent effects, such as ab initio molecular dynamics of condensed phases in conjunction with Travis analysis [9]. These procedures can be used to explore the IR [10], Raman [11] and optical activities [12] of various systems; however, this approach was beyond the scope of our work because normal mode description is indirect and is not practically applicable for flexible molecules.

For PhEA, fundamental vibrations were the main signals in the Raman spectra. For PIP, fundamental and combination bands were the main signals in the Raman spectra, as shown in Figure S14 and S15.

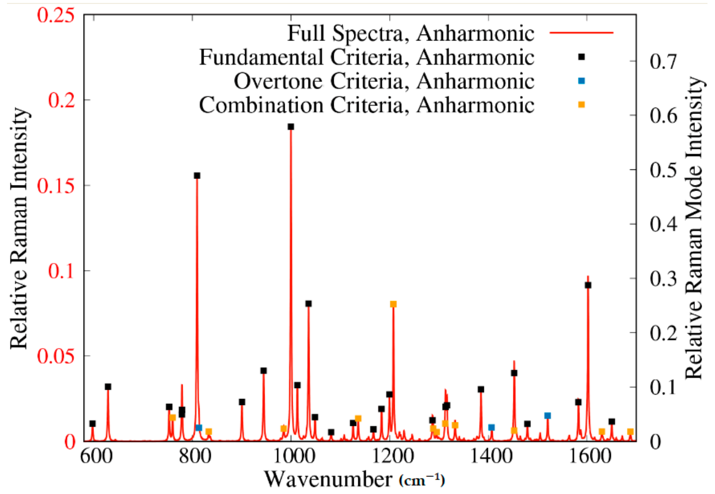

Figure S14. Raman spectra of PhEA constructed using all VPT2 data (fundamental, overtone and combination band wavenumbers and intensities) with Lorentzian function broadening (half-maximum bandwidth of 2 cm⁻¹for all normal modes (left axis). The ab initio intensities only for greater contribution normal modes (criteria) (right axis).

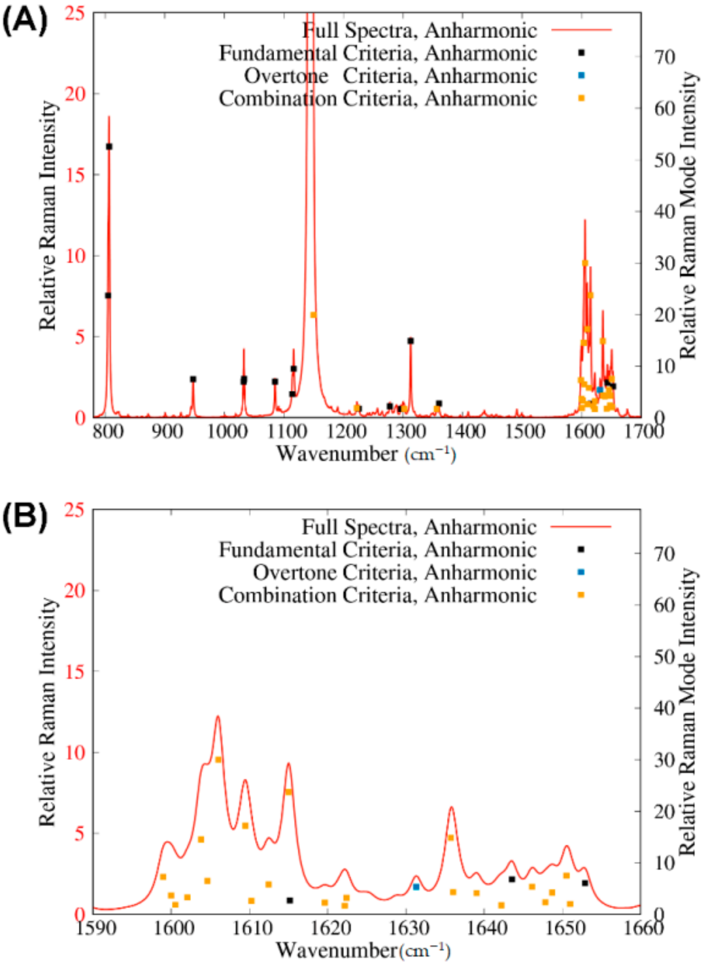

Figure S15. (A) Raman spectra of PIP constructed using all VPT2 data (fundamental, overtone and combination band wavenumbers and intensities) with Lorentzian function broadening (half-maximum bandwidth of 2 cm⁻¹) for all normal modes (left axis). The ab initio intensities only for greater contribution normal modes (criteria) (right axis); (B) Zoomed-in image of Figure S15(A) in the 1590–1660 cm⁻¹ wavenumber range.

S5. Comparison of theoretical estimation of Raman activity with experimental Raman spectra

Theoretical estimation of Raman activity was performed to assign vibrations and to explain superficial phenomena that occur upon CCD/P-PhEA-PIP interaction with AuNSs. Figure S16 and S17 compare the Raman spectra obtained by two different levels of theoretical estimation with the experimental Raman spectra.

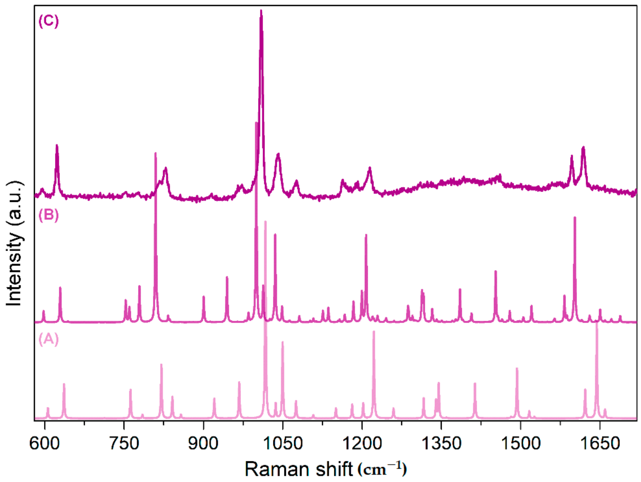

Figure S16. Theoretical estimation of Raman activity by (A) B3LYP harmonic and (B) VPT2 total anharmonic and (C) experimental spectra of PhEA.

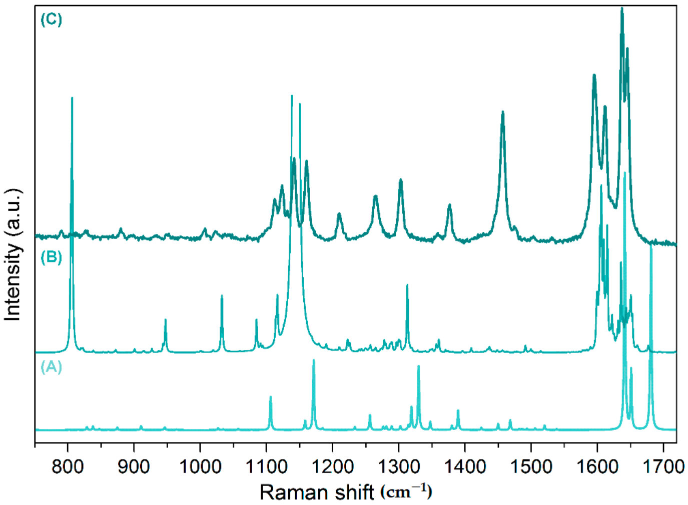

Figure S17. Theoretical estimation of Raman activity by (A) B3LYP harmonic and (B) VPT2 total anharmonic and (C) experimental spectra of PIP.

References

1. Strohalm, M.; Hassman, M.; Košata, B.; Kodíček, M. mMass data miner: An open source alternative for mass spectrometric data analysis. *Rapid Commun. Mass Spectrom.* **2008**, *22*, 905–908, doi:10.1002/rcm.3444.
2. Strohalm, M.; Kavan, D.; Novák, P.; Volný, M.; Havlíček, V. MMass 3: A cross-platform software environment for precise analysis of mass spectrometric data. *Anal. Chem.* **2010**, *82*, 4648–4651, doi:10.1021/ac100818g.
3. Niedermeyer, T.H.J.; Strohalm, M. mMass as a Software Tool for the Annotation of Cyclic Peptide Tandem Mass Spectra. *PLoS One* **2012**, *7*, e44913, doi:10.1371/journal.pone.0044913.
4. Wong, M.W. Vibrational frequency prediction using density functional theory. *Chem. Phys. Lett.* **1996**, *256*, 391–399, doi:10.1016/0009-2614(96)00483-6.
5. Andrade, S.G.; Gonçalves, L.C.S.; Jorge, F.E. Scaling factors for fundamental vibrational frequencies and zero-point energies obtained from HF, MP2, and DFT/DZP and TZP harmonic frequencies. *J. Mol. Struct. THEOCHEM* **2008**, *864*, 20–25, doi:10.1016/j.theochem.2008.05.025.
6. Iron, M.A.; Gropp, J. Using Cost-Effective Density Functional Theory (DFT) to Calculate Equilibrium Isotopic Fractionation for Reactions Involving Large Organic Molecules. *ChemRxiv* **2019**, doi:https://doi.org/10.26434/chemrxiv.8063654.v1.

7. Kim, K.; Jordan, K.D. Comparison of density functional and MP2 calculations on the water monomer and dimer. *J. Phys. Chem.* **1994**, *98*, 10089–10094, doi:10.1021/j100091a024.
8. Ravichandran, L.; Banik, S. Performance of different density functionals for the calculation of vibrational frequencies with vibrational coupled cluster method in bosonic representation. *Theor. Chem. Acc.* **2018**, *137*, 1, doi:10.1007/s00214-017-2177-9.
9. Brehm, M.; Thomas, M.; Gehrke, S.; Kirchner, B. TRAVIS—A free analyzer for trajectories from molecular simulation. *J. Chem. Phys.* **2020**, *152*, 164105, doi:10.1063/5.0005078.
10. Thomas, M.; Brehm, M.; Hollóczki, O.; Kelemen, Z.; Nyulászi, L.; Pasinszki, T.; Kirchner, B. Simulating the vibrational spectra of ionic liquid systems: 1-Ethyl-3-methylimidazolium acetate and its mixtures. *J. Chem. Phys.* **2014**, *141*, 024510, doi:10.1063/1.4887082.
11. Brehm, M.; Thomas, M. Computing Bulk Phase Resonance Raman Spectra from ab Initio Molecular Dynamics and Real-Time TDDFT. *J. Chem. Theory Comput.* **2019**, *15*, 3901–3905, doi:10.1021/acs.jctc.9b00512.
12. Brehm, M.; Thomas, M. Computing Bulk Phase Raman Optical Activity Spectra from ab initio Molecular Dynamics Simulations. *J. Phys. Chem. Lett.* **2017**, *8*, 3409–3414, doi:10.1021/acs.jpclett.7b01616.
